# The cellular and molecular targets of natural products against metabolic disorders: a translational approach to reach the bedside

**DOI:** 10.1002/mco2.664

**Published:** 2024-07-24

**Authors:** Xiaofei Shen, Hongling Yang, Yang Yang, Xianjun Zhu, Qingxiang Sun

**Affiliations:** ^1^ TCM Regulating Metabolic Diseases Key Laboratory of Sichuan Province Hospital of Chengdu University of Traditional Chinese Medicine Chengdu University of Traditional Chinese Medicine Chengdu China; ^2^ Department of Nephrology and Institute of Nephrology Sichuan Provincial People's Hospital, School of Medicine, University of Electronic Science and Technology of China, Sichuan Clinical Research Centre for Kidney Diseases Chengdu China; ^3^ Department of Respiratory and Critical Care Medicine Sichuan Provincial People's Hospital University of Electronic Science and Technology Chengdu China; ^4^ The Sichuan Provincial Key Laboratory for Human Disease Gene Study, Center for Medical Genetics Sichuan Provincial People's Hospital University of Electronic Science and Technology Chengdu China

**Keywords:** glucose/lipid metabolism, insulin resistance, metabolic disorders, metabolic inflammation, natural products, target identification

## Abstract

Metabolic disorders, including obesity, dyslipidemia, diabetes, nonalcoholic fatty liver disease, and metabolic syndrome, are characterized by insulin resistance, abnormalities in circulating cholesterol and lipid profiles, and hypertension. The most common pathophysiologies of metabolic disorders are glucose/lipid metabolism dysregulation, insulin resistance, inflammatory response, and oxidative stress. Although several agents have been approved for the treatment of metabolic disorders, there is still a strong demand for more efficacious drugs with less side effects. Natural products have been critical sources of drug research and discovery for decades. However, the usefulness of bioactive natural products is often limited by incomplete understanding of their direct cellular targets. In this review, we highlight the current understanding of the established and emerging molecular mechanisms of metabolic disorders. We further summarize the therapeutic effects and underlying mechanisms of natural products on metabolic disorders, with highlights on their direct cellular targets, which are mainly implicated in the regulation of glucose/lipid metabolism, insulin resistance, metabolic inflammation, and oxidative stress. Finally, this review also covers the clinical studies of natural products in metabolic disorders. These progresses are expected to facilitate the application of these natural products and their derivatives in the development of novel drugs against metabolic disorders.

## Graphical abstract

Metabolic disorders seriously endanger the health of human beings. However, there are no approved drugs specific for metabolic disorders. Recently, sever natural products have been demonstrated to alleviate metabolic disorders, and also used as chemical probes to identify their functional targets, which are involved in insulin resistance, glycolipid metabolism, and metabolic inflammation. These developments may facilitate the discovery of natural product‐based first‐in‐class therapeutics against metabolic disorders.

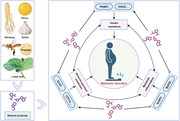

## 1 INTRODUCTION

Metabolic disorders are conditions in which the body does not properly process and distribute macronutrients such as carbohydrates, fat, and protein. It can be caused by inherited gene mutations and environmental factors such as excess nutrition or lack of exercise.^1^, ^2^ The most common metabolic disease is diabetes, a disease characterized by high blood glucose levels. Type 1 diabetes accounts for only approximately 5% of all diabetic patients and can be treated with insulin infusions.^3^ Type 2 diabetes (T2D) is unresponsive to insulin therapy (insulin resistant) and represents a major clinical challenge.^4^

**FIGURE 1:**
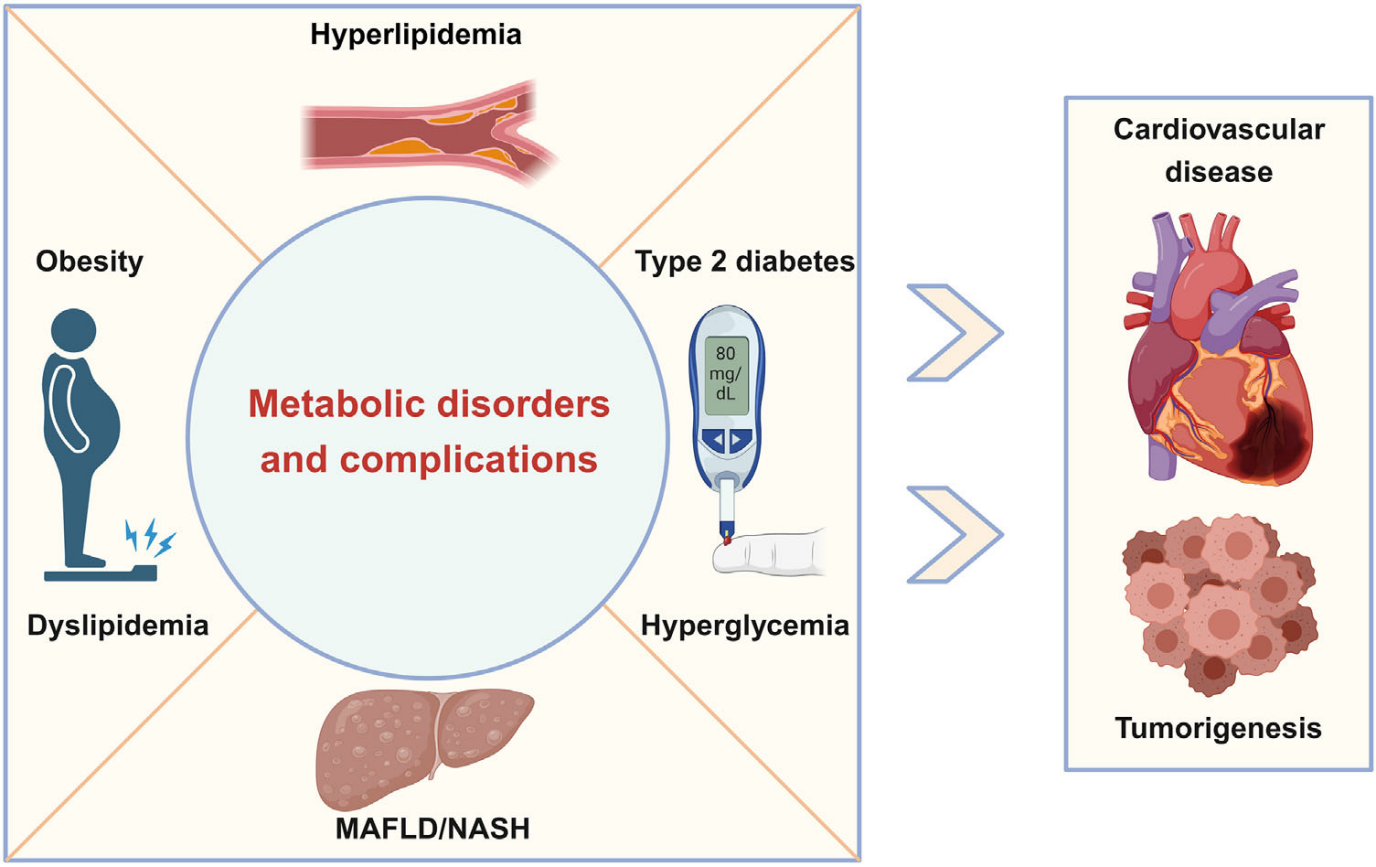
Metabolic disorders and its complications. Metabolic disorders mainly include hyperlipidemia, obesity, MAFLD/NASH, and T2D and its complications, which greatly increase the risk of cardiovascular and tumor diseases.

In most metabolic disorders, a lack of insulin or insulin resistance results in a high concentration of blood glucose, known as hyperglycemia. Over time, hyperglycemia causes damage to many organs, especially large and small blood vessels, to trigger diabetic complications.^5^ Macrovascular complications include hypertension and atherosclerosis, which can greatly increase the risk of developing conditions such as coronary heart disease, cardiomyopathy, and stroke.^6^ Microvascular complications include diabetic neuropathy, retinopathy, nephropathy, and so on.^7^ Diabetes‐induced cardiovascular disease is the leading cause of death in patients with diabetes.^8^ Moreover, excess glucose can be converted to lipids and stored in different tissues, fueling the development of obesity and metabolic dysfunction‐associated fatty liver disease (MAFLD, formerly known as non‐alcoholic fatty liver disease)/nonalcoholic steatohepatitis (NASH).^9^ In addition to causing damage to many organs and increasing the risk of lethal cardiovascular diseases, metabolic disorders increase the risk of certain cancers, such as liver, colorectal, breast, and pancreatic cancers.^10^, ^11^ Furthermore, metabolic disorders can cause varying degrees of physical discomfort for patients, limiting daily activities and increasing medical burden.^12^ In addition, metabolic disorders affect people of all ages and have been on the rise over the past few decades.^13^ Therefore, metabolic disorders represent a group of disorders associated with various interrelated pathological conditions including hyperlipidemia, obesity, hyperglycemia, MAFLD/NASH, T2D, and insulin resistance, that, when occurring together, strongly increase the incidence and mortality of cardiovascular diseases and cancers (Figure 1).^14^

Although several types of drugs, such as metformin, thiazolidinediones, dipeptidyl peptidase 4 inhibitors, and α‐glucosidase inhibitors, have been used for the clinical treatment of metabolic disorders, more efficacious drugs with less side effects are needed to treat metabolic disorders.^15^, ^16^, ^17^, ^18^, ^19^ In addition to medication treatment, current treatment options also include lifestyle changes such as exercise and diet. In severe cases of obesity, bariatric surgery in which part of the stomach or intestine is removed may be used to reduce weight.^20^, ^21^ Despite this, these diseases remain poorly controlled for multiple reasons, including patient compliance, access to healthcare, and lack of prevention strategies.

Natural products possess unsurpassed structural and chemical diversity and are the best sources of lead compounds and new drugs.^22^ However, the clinical translation of natural products still faces many hurdles, especially given the incomplete understanding of their direct functional targets.^23^ Therefore, target identification/validation is crucial for the development and utilization of natural products and the discovery of new drugs.^24^, ^25^ Recently, a series of natural products have been confirmed to exhibit robust protective effects against metabolic disorders.^26^, ^27^ Some of these natural compounds have been used as chemical probes to reveal their potential cellular targets and molecular mechanisms of action against metabolic disorders. However, there is no systematic review on the progress of natural products with promising therapeutic benefits as well as their functional targets against metabolic disorders.

In this review, we summarize the pathophysiological mechanisms of metabolic disorders and recent advances in understanding the protective effects of natural products against metabolic disorders in preclinical models. We then focused mainly on the direct functional targets and underlying mechanisms of these natural products. Finally, we also describe the current clinical applications and limitations of these natural products in metabolic disorders, aiming to facilitate the discovery of natural product‐based therapeutics for metabolic disorders.

## 2 THE FUNDAMENTAL PATHOPHYSIOLOGICAL MECHANISMS OF METABOLIC DISORDERS

**FIGURE 2:**
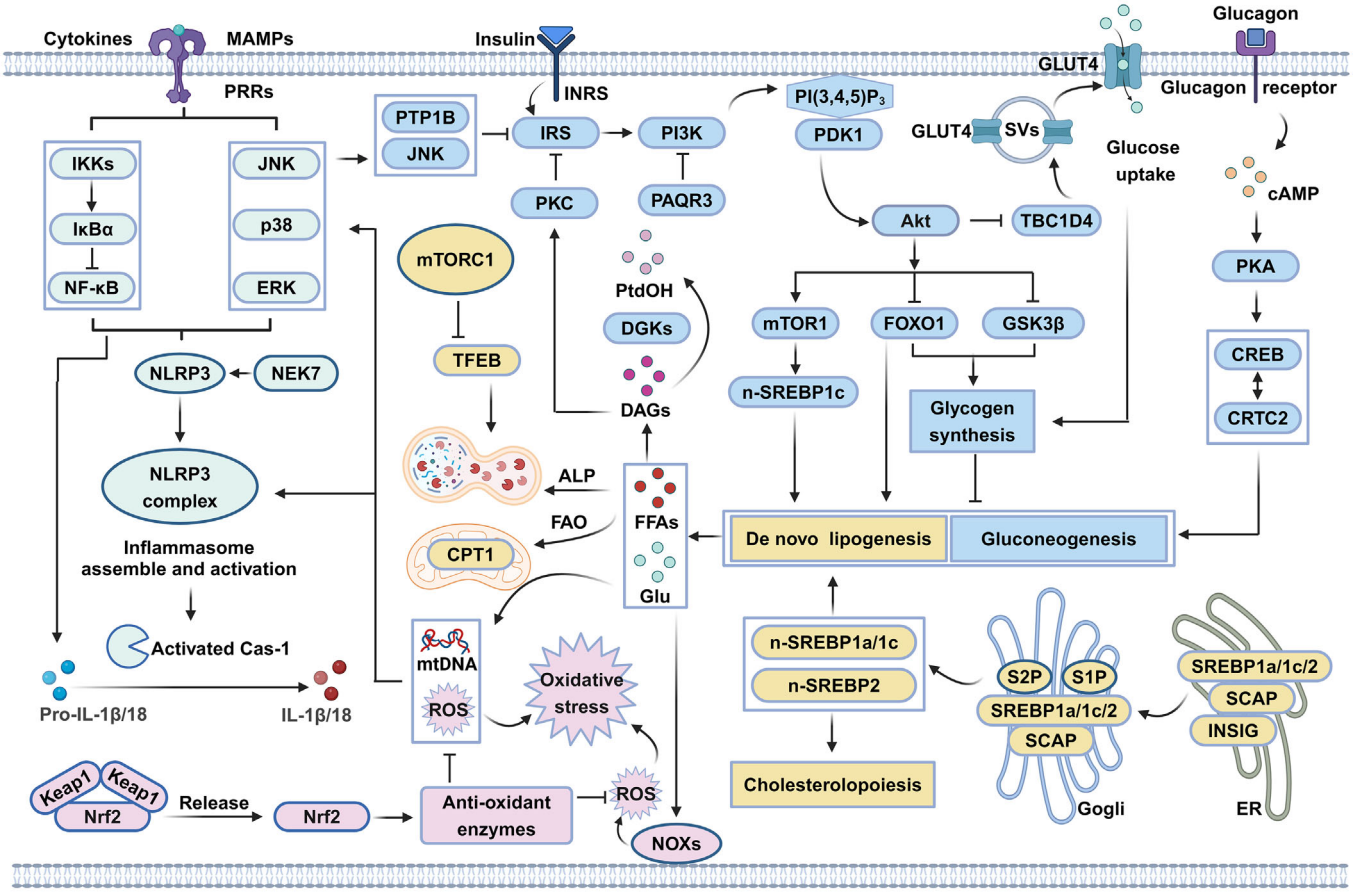
The molecular pathogenesis of metabolic disorders. Insulin binds to the insulin receptor and activates insulin receptor substrate (IRS), which recruits PI3K and activates Akt. Activated Akt then phosphorylates a series of substrates, including GSK3, mTOR1, FOXO1, and TBC1D4, which are involved in glycogen synthase, lipogenesis, and storage vesicles (SVs)‐mediated GLUT4 translocation. The insulin signaling pathway thus controls glucose levels. Furthermore, glucagon can also promote hepatic gluconeogenesis, glucose output, and lipogenesis by activating the cAMP–PKA–CREB/CRTC2 signaling axis. Moreover, SREBPs are a family of transcription factors that activate genes encoding enzymes required for the synthesis of cholesterol and fatty acids. SREBPs are controlled by multiple mechanisms involving proteolytic activation, subcellular localization, and transcriptional activity. Excess lipids can be eliminated by carnitine palmitoyltransferase 1 (CPT1)‐mediated fatty acid β‐oxidation (FAO) and TFEB‐mediated autophagy–lysosomal pathway (ALP). However, metabolism‐associated molecular patterns (MAMPs) derived from the overproduction of glucose and lipids can further activate several proinflammatory signaling pathways, such as the NF‐κB, MAPK, and NLRP3 pathways, thereby impairing the insulin‐mediated PI3K–Akt signaling pathway. Furthermore, several negative regulators such as PTP1B, PKC, and PAQR3 can also impair insulin signaling, thereby resulting in insulin resistance and hyperglycemia. In addition, metabolic overload causes ROS production through NOXs activation and mitochondrial uncoupling, which results in oxidative stress and destruction of bioactive macromolecules. Host cells can also activate the Nrf2 signaling axis to induce the expression of antioxidant genes. Cas‐1, caspase‐1; ERK, extracellular regulated protein kinases; Glu, glucose; IκBα, NF‐κB inhibitor alpha; PI(3,4,5)P_3_, phosphatidylinositol 3,4,5‐trisphosphate; PtdOH, phosphatidic acid.

Generally, the pathogenesis of metabolic disorders is multifactorial, which are involved in insulin resistance, disordered lipid metabolism, inflammation, oxidative stress, and so on (Figure 2). A deeper understanding of the underlying mechanisms of metabolic disorders is thus essential to the treatment of these diseases using natural products targeting specific target proteins.

### 2.1 Insulin resistance

Insulin resistance is a hallmark of a range of metabolic disorders, including obesity, T2D, cardiovascular diseases, and MAFLD.^28^, ^29^ Insulin resistance precedes the onset of diabetes and obesity and progressively worsens over the course of the disease.^30^ To maintain healthy blood glucose levels, β cells produce more insulin to compensate for resistance, resulting in elevated blood insulin levels, a condition known as hyperinsulinemia.^30^ Hyperinsulinemia always coexists with insulin resistance until β cells are unable to produce the amount of insulin needed to balance the rising insulin resistance, at which point blood glucose levels rise and symptoms of glucose intolerance develop. Hyperinsulinemia can cause insulin resistance in lean individuals and induce obesity through the action of insulin on lipogenesis.^31^, ^32^ Therefore, insulin resistance and hyperinsulinemia are key to treating metabolic disorders,^28^, ^29^, ^31^, ^32^ and understanding how they develop offers opportunities to prevent and combat metabolic disorders.

Under fasting conditions, glucose is produced mainly by the liver through glycogenolysis and gluconeogenesis, while fatty acids are produced from fat tissue through lipolysis.^33^ The resulting glucose and lipids are released into the blood to provide energy for other organs, such as the muscles and brain. Glucagon produced under fasting conditions can enhance hepatic gluconeogenesis and glucose output by activating cyclic adenosine monophosphate‐protein kinase A (cAMP‐PKA) and downstream cAMP‐response element binding protein/CREB regulated transcriptional coactivator 2 (CREB/CRTC2) transcriptional activity.^34^ Under fed conditions, a high concentration of blood glucose stimulates pancreatic β cells to secrete insulin into the blood.^35^ Circulating insulin inhibits glucose release from the liver and promotes glucose uptake by the liver, muscle, and adipose tissue.^36^ In addition, insulin inhibits lipolysis in adipocytes and promotes lipogenesis in the liver and adipocytes.^37^ This sophisticated system relies on proper insulin secretion from β cells and intact insulin signaling in other tissues.

Insulin signaling can be divided into proximal and distal segments. The proximal segment begins with the binding of insulin to the insulin receptor (INSR), which activates the latter and phosphorylates the insulin receptor substrate (IRS). Phosphorylated IRS recruits phosphoinositide 3‐kinase (PI3K), which further recruits serine/threonine kinases such as pyruvate dehydrogenase kinase 1 (PDK1), mammalian target of rapamycin complex 2 (mTORC2), and protein kinase B (Akt) to the plasma membrane through membrane lipid modification.^30^, ^38^ Conversely, progesterone and adipoQ receptor 3 (PAQR3), a Golgi‐localized transmembrane protein, can inhibit insulin signaling by inhibiting PI3K subunit assembly.^39^ Protein tyrosine phosphatase 1B (PTP1B) inhibits insulin signaling through dephosphorylation of the insulin receptor and IRS.^40^

Recruited PDK1 and mTORC2 phosphorylate and activate Akt, a key node in insulin signaling that phosphorylates more than 100 substrates acting in the distal segment. Among those, phosphorylation of the substrate TBC1 domain family member 4 (TBC1D4), a GTPase‐activating protein that regulates endosomal trafficking, promotes translocation of the glucose transporter glucose transporter glucose transporter‐4 (GLUT4) to the plasma membrane and glucose uptake.^30^, ^38^ Phosphorylation of the substrate glycogen synthase kinase 3 (GSK3) inhibits its activity and stimulates the synthesis of glycogen from glucose. Phosphorylation of the substrates forkhead box O (FOXO) proteins lead to the exclusion of FOXO proteins from the nucleus, thus reducing the transcription of gluconeogenic genes.^30^, ^38^ Akt can also phosphorylate the mammalian target of rapamycin complex 1 (mTORC1)–ribosomaiprotein S6 kinase (S6K) axis, which in turn leads to the activation of sterol regulatory element‐binding protein 1c (SREBP1c), a critical transcription factor for genes involved in lipid synthesis and uptake.^30^, ^38^, ^41^ While these and other insulin signaling mechanisms are well documented, the regulation of this signaling network is complex and not fully understood.

Consistent with the complexity of insulin signaling, past efforts have elucidated many mechanisms that are implicated in insulin resistance, that is, genetic abnormalities or environmental stress (overeating) dysregulates molecules involved in proximal and distal insulin signaling.^42^ Factors known to interfere with insulin signaling and trigger insulin resistance include hyperlipidemia, inflammation, and oxidative stress. These factors are highly interlinked and often coexist in metabolic disorders, as we review in the following sections.

### 2.2 Disordered lipid metabolism

Hyperlipidemia is characterized by high serum levels of fatty acids, triglycerides, and cholesterol and contributes to the development of insulin resistance and metabolic disorders.^43^ In mammals, the biosynthesis of fatty acids, triglycerides, and cholesterol is tightly regulated by the transcriptional control of genes involved in this process by several transcription factors, including SREBPs, liver X receptor α (LXRα), and peroxisome proliferator‐activated receptor γ (PPARγ).^44^, ^45^, ^46^ In particular, SREBPs are master transcription factors that regulate lipogenesis and cholesterogenesis. Among its three isoforms, SREBP‐1a and SREBP‐1c preferentially enhance the transcription of genes required for fatty acid synthesis, whereas SREBP‐2 preferentially promotes the induction of genes required for cholesterol synthesis and uptake.^45^

Excess blood glucose can be stored as lipids in fat tissues, and this process is thought to constitute a defense mechanism against hyperglycemia to prevent diabetes‐related symptoms. Genomic analysis has shown that a large proportion of T2D genetic defects are associated with adipose tissue dysfunction.^47^ These genes are involved in adipocyte differentiation or adipogenesis, among which PPARγ is a master regulator of adipogenesis.^48^ Pharmacological activation of PPARγ stimulates adipose tissue expansion and muscle insulin sensitivity in T2D patients.^49^

It is increasingly recognized that the inability of adipose tissue to properly store lipids leads to more lipids entering the circulation, being deposited in other tissues, and inducing insulin resistance in those tissues.^50^ For example, the accumulation of lipids in the pancreas can lead to β‐cell inflammation and secretion defects.^51^ Excess lipids can be converted to diacylglycerols (DAGs) and ceramide, notorious molecules that cause insulin resistance, dyslipidemia, and ultimately cell death in different tissues. Conversely, the intracellular levels of DAGs are strictly regulated by diacylglycerol kinases (DGKs), which alleviate DAGs‐induced insulin resistance.^52^ Furthermore, overproduction of lipids in nonfat tissues via hyperactive lipid biosynthesis may damage those tissues, leading to inflammation and insulin resistance.^53^, ^54^

Upregulation of thermogenesis can reduce the burden of fat storage. Uncoupling protein 1 (UCP1), activated through the PKA–p38 mitogen‐activated protein kinase (MAPK) signaling axis, mediates nonshivering thermogenesis in brown and beige adipocytes by dissipating the energy of the mitochondrial proton as heat.^55^, ^56^ Fatty acid β‐oxidation (FAO) is another metabolic pathway that contributes to energy production and thermogenesis.^57^ Promoting the expression and activity of enzymes involved in FAO, such as carnitine palmitoyltransferase 1 (CPT1), has been confirmed to enhance insulin sensitivity in mice.^58^ In addition to thermogenesis and FAO, the autophagy–lysosomal pathway (ALP)‐mediated degradation of accumulated lipids also participates in lipid homeostasis and is strictly regulated by mTORC1 and transcription factor EB (TFEB) signaling.^59^, ^60^ An alternative route to reduce lipid storage burden is to inhibit cholesterol and fatty acid biosynthesis, a process regulated by the transcription factor SREBPs.^44^ SREBPs are retained in the endoplasmic reticulum (ER) by forming a complex with SREBP cleavage‐activating protein (SCAP) and insulin‐induced gene (INSIG) at high cholesterol levels in normal cells, but they are upregulated in many metabolic disorders, leading to uncontrolled production of lipids and insulin resistance.^61^

### 2.3 Inflammation

A chronic low‐grade inflammatory response is a hallmark of several metabolic disorders, such as obesity, T2D, and its complications, atherosclerosis, and MAFLD.^62^ Chronic inflammation in metabolic disorders, termed “metabolic inflammation”, is initiated by metabolism‐associated molecular patterns (MAMPs) derived from metabolic overload, such as free fatty acids (FFAs), oxidized low‐density lipoprotein (ox‐LDL), glucose, advanced glycation end products (AGEs), and cholesterol crystals. These MAMPs can be recognized by several pattern recognition receptors (PRRs) such as Toll‐like receptor 4 (TLR4) in immune cells, including macrophages, to activate a series of proinflammatory signaling cascades, such as the myeloid differentiation primary response protein‐88 (MyD88)‐mediated nuclear factor kappa‐B (NF‐κB) and MAPK pathway, thereby promoting the release of proinflammatory factors.^63^ Among these cytokines, interleukin 1β (IL‐1β), a potent proinflammatory cytokine, is critical for β‐cell dysfunction and defective insulin secretion.^64^ In addition to NF‐κB and MAPK, the nucleotide‐binding oligomerization domain‐like receptor thermal protein domain associated protein 3 (NLRP3) inflammasome, a multimeric complex consisting of NLRP3, apoptosis‐associated speck‐like protein containing CARD (ASC), and caspase‐1, is also responsible for IL‐1β production.^65^ Deletion or inhibition of IL‐1β has been confirmed to reduce hyperglycemia in mice and humans.^62^

Furthermore, inflammation is associated not only with β‐cell dysfunction but also with insulin resistance in other organs, including the liver, adipose tissue, and muscle tissue.^64^ A variety of danger signaling molecules and cytokines, such as IL‐1β and tumor necrosis factor‐α (TNF‐α), can activate a series of intracellular signaling cascades, especially those involving the inhibitor of NF‐κB kinase β (IKKβ) and c‐Jun N‐terminal kinase (JNK), which further induce the serine phosphorylation of IRS1/2 and thereby impairing the activation of insulin signaling.^66^, ^67^ However, inflammatory marker levels are unchanged in the early stages of human T2D, and obesity‐induced insulin resistance in mice usually precedes macrophage accumulation and tissue inflammation. These results suggest that inflammation may not be the cause of insulin resistance pathogenesis but rather may mediate the progression of insulin resistance.^68^ Nonetheless, the suppression of inflammation has been actively explored clinically for the treatment and/or improvement of metabolic disorders.

Like in islets, chronic metabolic overload also results in damage to biomacromolecules and organelles, especially mitochondria, which are likely the major initiating source of tissue inflammation.^69^ For example, impaired mitochondria can release abundant mitochondrial DNA (mtDNA) and mitochondrial reactive oxygen species (mtROS), which are regarded as damage‐associated molecular patterns, and in turn activate a series of downstream proinflammatory signaling pathways, such as the NLRP3 inflammasome and NF‐κB pathways.^70^ PTEN‐induced kinase 1 (PINK1)‐Parkin‐mediated mitophagy, or mitochondrial autophagy, can effectively eliminate damaged mitochondria and inhibit the release of mtDNA and mtROS.^71^ However, in metabolic disorders such as MAFLD, this pathway is often impaired due to the suppression of the PINK1–Parkin axis and/or overactivation of mTORC1 and subsequent inactivation of TFEB, the master transcriptional regulator of autophagy.^72^ Therefore, enhancing mitophagy/autophagy has been demonstrated to reduce inflammation and represents a new strategy for the treatment of metabolic disorders.^71^, ^72^

### 2.4 Oxidative stress

ROS are constantly and inevitably generated from mitochondria in all cells. Electrons can leak from the electron transport chain and bind oxygen to form superoxide (O_2_^•^) and hydrogen peroxide (H_2_O_2_), the major ROS species in cells. These ROS species are highly reactive and, if not eliminated rapidly, can react with adjacent lipids, proteins, and DNA, causing damage to these cellular components.^73^, ^74^ To prevent damage accumulation, cells use a variety of endogenous antioxidants, such as catalase (CAT), superoxide dismutase (SOD), glutathione peroxidase (GPX), thioredoxins, vitamin C/E, and reduced glutathione (GSH), to eliminate or maintain low ROS levels.^75^ An imbalance between ROS production and antioxidant capacity generates oxidative stress and is observed in many tissues with metabolic disorders.^74^ Hyperglycemia plays a key role in ROS generation and oxidative stress by activating multiple pathways, including the polyol pathway, the protein kinase C (PKC) pathway, and the AGEs pathway.^76^, ^77^ In the polyol pathway, elevated glucose levels activate aldose reductase, which converts glucose to sorbitol, but this process consumes nicotinamide adenine dinucleotide phosphate (NADPH) and inhibits downstream GSH synthesis. Furthermore, hyperglycemia‐induced DAGs and AGEs can activate the PKC and receptor for AGEs pathways, respectively, thereby activating NADPH oxidases (NOXs), the major ROS producers in cells.^76^, ^77^ Moreover, excess ROS also trigger robust immune and inflammatory responses by activating the NF‐κB pathway, the NLRP3 inflammasome, and MAPK. While ROS can trigger and propagate inflammation, inflammation itself can also lead to ROS production.^78^ Thus, inflammation and oxidative stress often work hand in hand in individuals with normal physiology and metabolic disorders, forming a positive feedback loop.^79^

Oxidative stress is associated with β‐cell dysfunction, in part by inducing long‐term inflammation in these cells. Alternatively, β cells often express fewer antioxidant enzymes and are more susceptible to oxidative stress. Oxidative damage to organelles such as mitochondria, the ER, and the Golgi apparatus impairs normal β‐cell functions, including insulin production.^80^ Nuclear factor erythroid 2‐related factor 2 (Nrf2) is a master transcription factor that binds to antioxidant response elements in the promoter regions of genes encoding various antioxidant enzymes and detoxification proteins. The survival, function, and proliferation of β cells critically depend on Nrf2‐mediated ROS control.^81^

Furthermore, oxidative stress is known to impair insulin signaling through multiple mechanisms. For example, oxidative stress in muscle cells leads to partial loss of IRS1 and IRS2 in a p38 MAPK‐dependent manner.^82^ In addition, ROS directly oxidize and inactivate insulin receptors or dephosphorylate and inactivate Akt through the activation of phosphatases.^83^ In conclusion, studies have shown that oxidative stress plays a key role in metabolic disorders, mainly by inducing inflammation, β‐cell dysfunction, and insulin resistance.^78^, ^79^

### 2.5 Others

Research has shown that fat cells also secrete several “good” signaling molecules, such as leptin and adiponectin.^84^ Leptin was the first identified adipokine (a signaling molecule from adipose tissue) and regulates energy balance in several ways, for example, by acting on the hypothalamus to reduce appetite or by “browning” white adipose tissue (WAT) to stimulate energy expenditure.^85^ However, similar to insulin resistance, leptin resistance is common in patients with metabolic diseases and is mainly driven by inflammation and ER stress in the hypothalamus.^85^ Adiponectin is another adipokine that enhances insulin sensitivity, reduces inflammation, and increases fatty acid oxidation, but it is often downregulated in metabolic disorders.^86^ Adiponectin exerts these effects by binding to its cell surface receptors, which then phosphorylate and activate adenosine 5′monophosphate‐activated protein kinase (AMPK) and downstream signaling pathways.^86^

In addition, as both metabolites and metabolic gene expression broadly exhibit circadian oscillations, metabolic processes are thus precisely regulated by the biological circadian clock.^87^ In humans, dysfunction of the circadian rhythm has been demonstrated to cause metabolic disorders such as glucose intolerance and hyperlipidemia.^88^ Metabolic deficiencies were also observed in mice with rhythmic disorders.^89^ For example, the Clock^Δ19/Δ19^ mutant, which harbors a dominant negative allele, was found to result in a series of metabolic disorders, including obesity, hyperlipidemia, hepatic steatosis, and hyperglycemia, in mice.^89^ Therefore, correcting rhythmic disorders also represents a potential strategy for treating metabolic disorders.^87^, ^88^

## 3 THE EFFECTS AND FUNCTIONAL TARGETS OF NATURAL PRODUCTS IN THE TREATMENT OF METABOLIC DISORDERS

**FIGURE 3:**
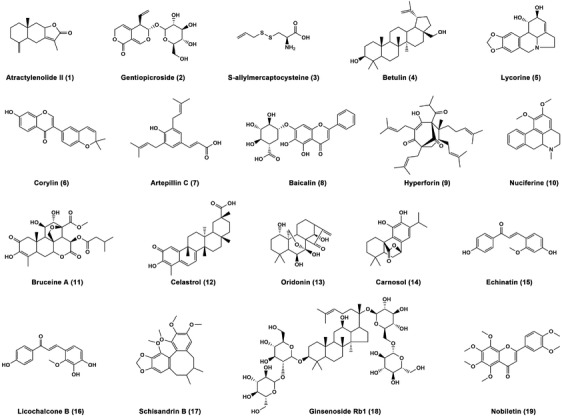
Chemical structures of the natural products discussed in this review. There are a total of 19 natural products, including sesquiterpene (**1**), iridoid (**2**), diterpenoids (**11**, **13**, and **14**), triterpenes (**4**, **12**, and **18**), flavonoids (**6**, **8**, and **19**), chalcones (**15** and **16**), alkaloids (**5** and **10**), phenols (**7** and **9**), sulfide (**3**), and lignanoid (**17**).

As mentioned above, the pathogenesis of metabolic disorders is insulin resistance, hyperlipidemia, metabolic inflammation, and oxidative stress.^14^ Despite lacking approved specific therapeutic drugs, numerous phytochemicals from diverse medicinal plants have exhibited therapeutic potentials for metabolic disorders in vitro and in vivo. In particular, some of those bioactive compounds have been used as probe for target identification. Herein, we summarize the improvement effects, molecular mechanisms, and functional targets of 19 natural products against metabolic disorders, including sesquiterpene, iridoid, diterpenoids, triterpenes, flavonoids, chalcones, alkaloids, phenols, sulfide, and lignanoid (Figure 3).

### 3.1 Natural products that directly restore the insulin signaling cascade

**FIGURE 4:**
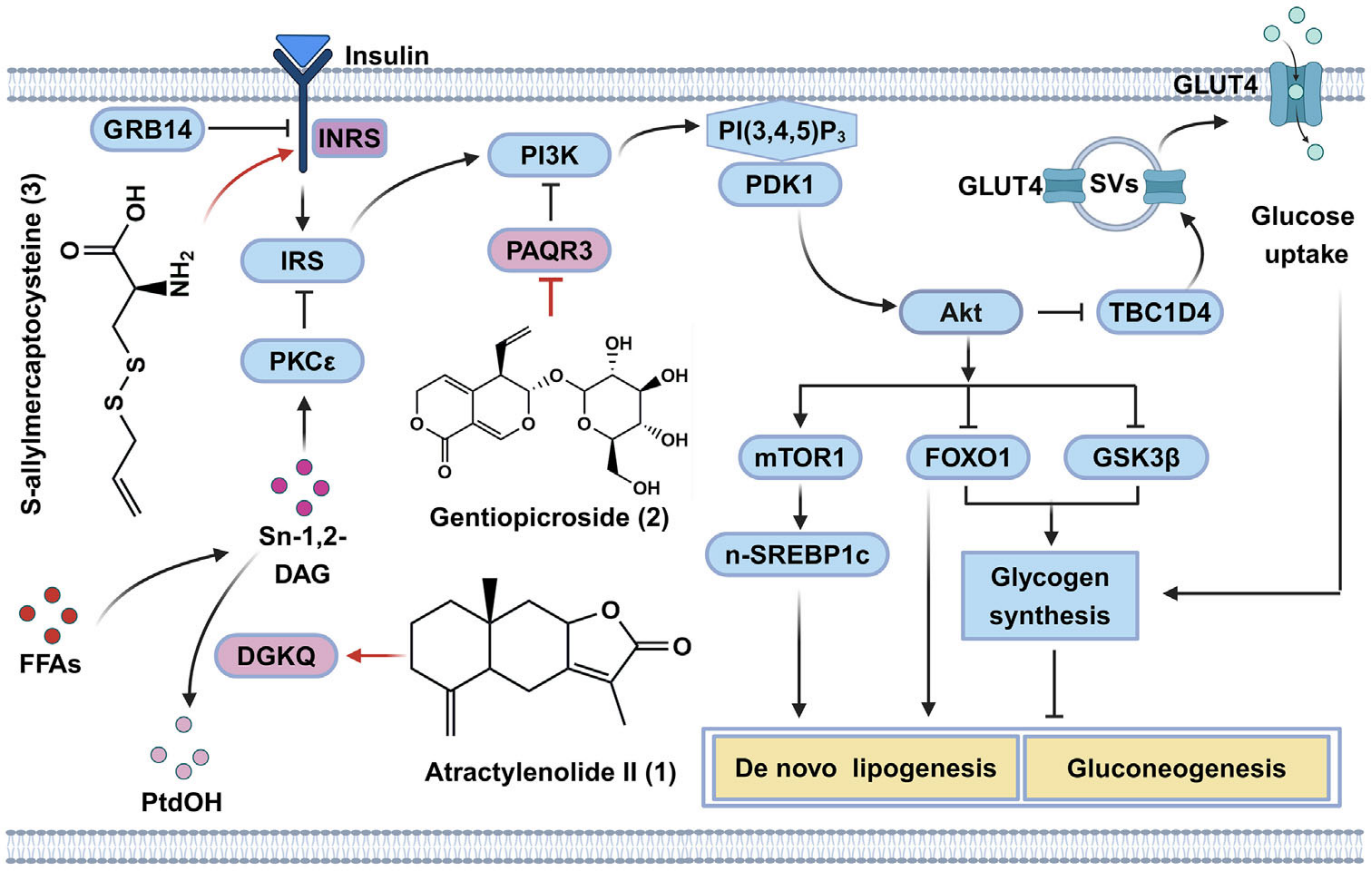
The functional targets and underlying mechanisms of natural compounds against insulin resistance. Among them, atractylenolide II (**1**) allosterically activates DGKQ, thereby relieving the inhibition of sn‐1,2‐DAG–PKCε signaling axis on insulin signaling. Gentiopicroside (**2**) directly binds to the negative regulator PAQR3, thus enhancing insulin signaling. Furthermore, S‐allylmercaptocysteine (**3**) binds to INSR, thereby disturbing the interaction between INSR and GRB14 to restore insulin signaling.

In this section, we discussed the therapeutic effects, molecular mechanisms, and functional targets of three natural products against metabolic disorders via directly restoring insulin signaling (Figure 4). Among them, atractylenolide II (**1**) directly activated DGKQ, the key enzyme negatively regulates insulin resistance via reducing cellular diacylglycerol (DAG). Gentiopicroside (**2**) acted as an inhibitor of PAQR3, a negative regulator of insulin signaling. S‐allylmercaptocysteine (SAMC; **3**) directly bound to insulin receptor INSR, thereby restoring insulin signaling.

#### 3.1.1 Atractylenolide II allosterically activates DGKQ to ameliorate obesity‐induced insulin resistance

Pathological elevation of DAGs, especially sn‐1,2‐DAG, is strongly associated with insulin resistance and T2D via direct activation of PKCε and PKCε‐mediated inactivation of PI3K–Akt signaling.^90^ Therefore, inhibiting the sn‐1,2‐DAG–PKCε signaling axis is considered a promising strategy for improving insulin resistance and metabolic disorders.^54^, ^90^

In 2023, Zheng et al.^91^ identified atractylenolide II (**1**), a natural sesquiterpenoid isolated from *Atractylodes macrocephala*, as the first small molecule inhibitor of hepatic sn‐1,2‐DAG–PKCε signaling axis. Atractylenolide II effectively rescued impaired insulin receptor kinase–PI3K–Akt signaling, thereby inactivating glycogen synthase kinase 3β (GSK3β) and promoting the phosphorylation and nuclear translocation of FOXO1, ultimately repressing gluconeogenesis, enhancing glycogenesis, and improving insulin resistance in palmitic acid (PA)‐stimulated HepG2 cells, diet‐induced obesity (DIO) mice, and ob/ob mice (genetically obese mice). Additionally, hepatic overexpression of PKCε or activation of PKCε by 8‐[2‐(2‐pentyl‐cyclopropylmethyl)‐cyclopropyl]‐octanoic acid (DCP‐LA) （ markedly reversed the atractylenolide II‐mediated improvement on insulin resistance in vitro and in vivo, suggesting the key role of the sn‐1,2‐DAG–PKCε signaling axis in the pharmacological potency of atractylenolide II.

Using activity‐based protein profiling strategy, diacylglycerol kinase theta (DGKQ), an enzyme that converts DAG into phosphatidate, was identified as the functional target of atractylenolide II. Interesting, atractylenolide II only directly bound to DGKQ, but not other DGKs family proteins such as DGKA, DGKE, and DGKZ.^91^ Furthermore, being different from atractylenolide II, its analogues such as atractylenolide I, 8β‐methoxyatractylenolide I, and atractylenolide III, partially or almost completely lost the binding ability to DGKQ. These results suggested a direct and specific interaction between DGKQ and atractylenolide II. Additionally, several amino acid residues including Ser193, Cys204, and Ser241 in the cysteine‐rich domain and Ala496, Phe497, and His498 in PH domain were essential for the binding of atractylenolide II to DGKQ. Upon noncovalent binding to these residues, atractylenolide II allosterically activated DGKQ, thereby reducing intracellular sn‐1,2‐DAG, ultimately inhibiting the PKCε signaling axis and improving insulin resistance. Conversely, DGKQ knockout almost completely abolished atractylenolide II‐mediated inhibition of the sn‐1,2‐DAG–PKCε signaling axis and insulin resistance.^91^ Additionally, extract of *A. macrocephala* has been demonstrated to enhance insulin sensitivity via activating PI3K–Akt signaling, which may be associated with the activation of atractylenolide II on DGKQ.^92^

Furthermore, Zheng et al.^91^ also found that the activity of DGKQ were decreased significantly in obese mice, which suggested that DGKQ might be a potential target for the development of antiobesity drugs. Although the expression of DGKQ is low in adipose tissue, atractylenolide II at a high dose (60 mg/kg) for 6 weeks also increased energy expenditure and weight loss by activating the AMPK1–PPARγ coactivator 1α (PGC1α)–UCP1 signaling axis in a DGKQ‐dependent manner.^91^ Taken together, these findings suggest that DGKQ is a valuable target for obesity related hepatosteatosis and insulin resistance. Furthermore, these results also propose that atractylenolide II is a novel allosteric activator of DGKQ and is a promising lead compound for improving obesity‐induced insulin resistance.

#### 3.1.2 Gentiopicroside directly binds PAQR3 and ameliorates glycolipid metabolism disorders

PAQR3, a seven transmembrane receptor located on the Golgi membrane, has been found to negatively regulate insulin‐mediated PI3K–Akt signaling by binding to p110α and disturbing the formation of the p110α–p85α PI3K complex.^39^ Therefore, the upregulation of PAQR3 is involved in insulin resistance and metabolic disorders, and PAQR3 represents a valuable target for combating metabolic diseases.^93^

In 2022, Xiao et al.^94^ reported that gentiopicroside (**2**), the main bioactive secoiridoid glycoside of *Gentiana manshurica* Kitagawa, not only decreased lipid synthesis and increased glucose utilization in PA‐stimulated HepG2 cells but also improved glycolipid metabolism in streptozotocin (STZ)/high‐fat diet (HFD)‐induced diabetic mice. These effects were mainly attributed to the restorative effect of these compounds on insulin‐induced PI3K–Akt signaling.

The authors further demonstrated that gentiopicroside could enhance the activation of the PI3K–Akt pathway by inhibiting the interaction between PAQR3 and p110a in PA‐treated HepG2 cells and liver tissues from STZ/HFD‐induced diabetic mice.^94^ Mechanistically, gentiopicroside facilitated the interaction of PAQR3 with damage specific DNA‐binding protein 2 (DDB2), an adaptor involved in the initiation of ubiquitination, by forming a ubiquitin ligase complex with DDB1 and cullin 4A, thereby promoting DDB2‐mediated ubiquitination of PAQR3 both in vitro and in vivo. Furthermore, gentiopicroside was found to directly bind to the N‐terminus of PAQR3 by forming hydrogen bonds with Leu40, Asp42, Glu69, Tyr125, and Ser129, thereby disrupting the interaction between PAQR3 and the PI3K catalytic subunit p110α, allowing PI3K–Akt signaling and insulin sensitivity.

Given that PAQR3 is implicated in the regulation of inflammation via activation of the NF‐κB pathway,^95^ the inhibitory effect of gentiopicroside on PAQR3 may also contribute to its previously reported anti‐inflammatory effect.^96^ These different effects of gentiopicroside may explain its antidiabetic activity and warrant further studies. Overall, gentiopicroside was identified as the first natural inhibitor of PAQR3 and could be used as an insulin sensitizer in the treatment of metabolic syndrome. Structural optimization of gentiopicroside to increase its affinity for PAQR3 is needed before clinical investigations.

#### 3.1.3 SAMC directly targets INSR to ameliorate ALD

Alcoholic liver disease (ALD) is caused by the excessive intake of ethanol and is characterized by steatosis, steatohepatitis, and even cirrhosis. Excessive ethanol intake disturbs insulin signaling, thereby leading to insulin resistance and subsequent hepatic lesions.^97^ Therefore, pharmacological activation of insulin signaling represents a potential treatment strategy for ALD.^98^

SAMC (**3**) is a water‐soluble ingredient isolated from aged garlic. Previously, SAMC was proven to ameliorate HFD‐induced MAFLD in rats^99^; however, the underlying mechanisms and functional targets of SAMC have not been identified. In 2021, Luo et al.^100^ reported that SAMC directly binds to the INSR protein by using cellular thermal shift assay (CETSA) and surface plasmon resonance (SPR) analysis. Molecular docking revealed that SAMC formed three hydrogen bonds with Arg1164, Lys1182, and Asp1183 of INSR. Furthermore, the hydrophobic interactions between SAMC and Met1171, Met1176, and Phe1186 also contributed to the binding of SAMC to INSR. These residues are located in the insulin receptor tyrosine kinase (IRTK) domain of INSR, which is utilized for the interaction of growth factor receptor‐bound protein 14 (GRB14), a negative regulator of insulin signaling. Therefore, the binding of SAMC to this interface may disturb the interaction between IRTK and GRB14, thereby sustaining insulin signal transduction.

SAMC not only inhibited ethanol/PA‐induced lipid accumulation and cell death in alpha mouse liver 12 (AML‐12) cells but also mitigated ethanol‐induced liver injury in mice. The hepatoprotective effect of SAMC was associated with its restoration of Akt–GSK3β signaling, which was abolished by INSR knockdown. In addition, 90 days of continuous treatment with SAMC at a dose of 300 mg/kg did not result in detectable adverse effects in normal mice.^100^ Taken together, these findings indicate that SAMC is an effective and safe hepato‐protective natural product against ALD through the promotion of insulin‐mediated Akt–GSK3β signaling via direct binding to ISNR.

### 3.2 Natural products that inhibit glycolipid synthesis

**FIGURE 5:**
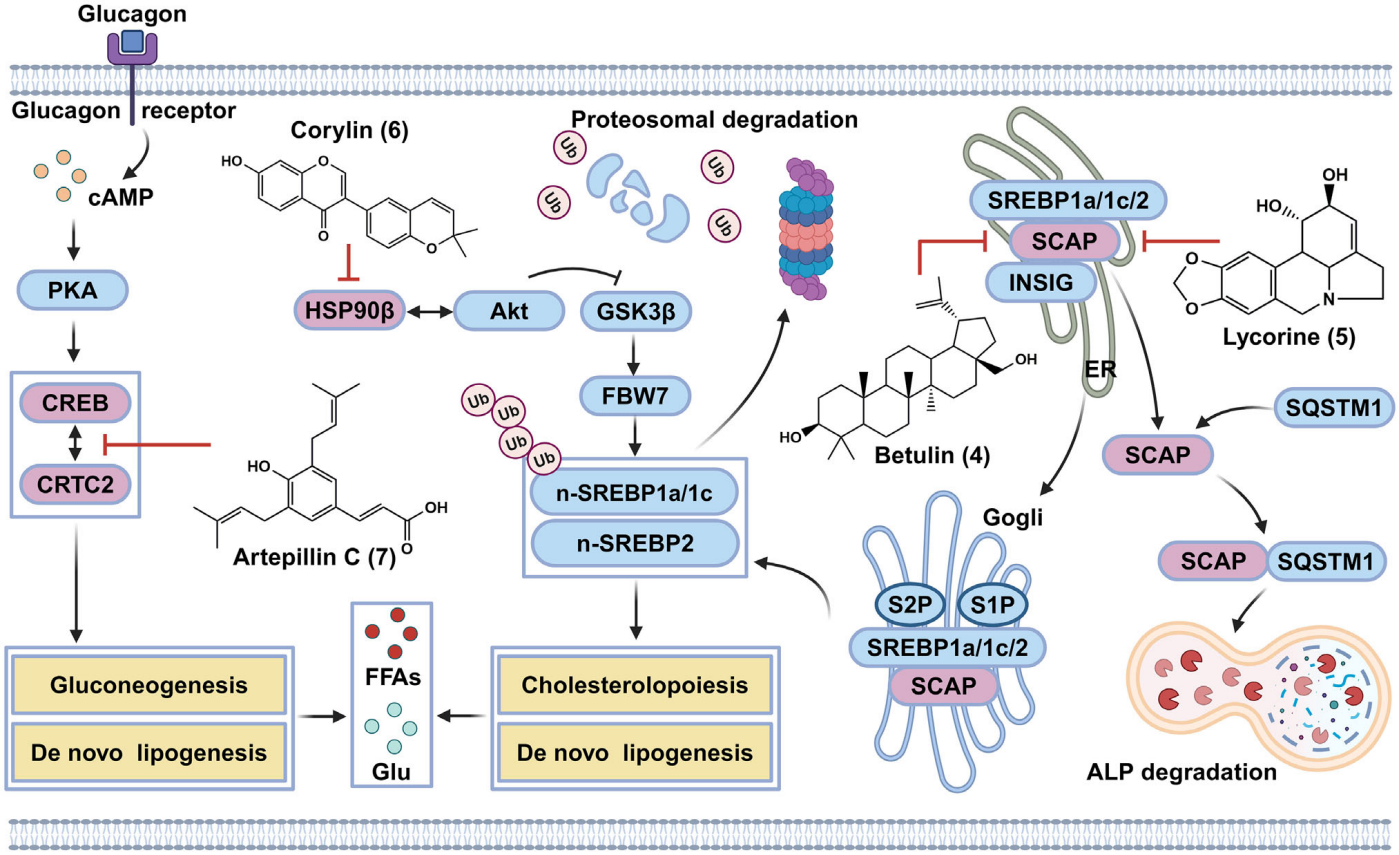
The functional targets and underlying mechanisms of natural products against hyperglycemia and hyperlipidemia. Among them, betulin (**4**) directly binds to SCAP and inhibits the maturation of SREBPs. Lycorine (**5**) directly binds to SCAP and induces SCAP degradation, thereby inhibiting SREBPs maturation. Corylin (**6**) inhibits SREBPs maturation via binding to HSP90β. Artepillin C (**7**) directly binds to CREB, thereby inhibiting CREB–CRTC2 interaction and CREB/CRTC2‐mediated gluconeogenesis and lipogenesis. UB, ubiquitin.

Herein, the improvement effects, underlying mechanisms, and functional targets of four natural compounds against metabolic disorders via inhibiting glycolipid synthesis were reviewed in this section, which were presented in Figure 5. Betulin (**4**) and lycorine (**5**) directly bound to the SREBP cleavage activating protein SCAP, thereby inhibiting maturation of the SREBP transcription factors. Corylin (**6**) functioned as a HSP90β inhibitor, thereby promoting degradation of mature SREBPs. Artepillin C (**7**) was found to inhibit gluconeogenesis and lipogenesis via interacting with CREB transcription factor.

#### 3.2.1 Betulin inhibits the SREBP pathway by interacting with SCAP

Newly synthesized precursors of SREBPs (pre‐SREBPs) are inserted into the ER membrane, where SREBPs interact with SCAP. In low sterol cells, the SREBP–SCAP complex is transported from the ER to the Golgi apparatus, where the N‐terminus of SREBP is cleaved by site‐1 protease (S1P) and site‐2 protease (S2P) and subsequently released from the Golgi membrane. The released n‐SREBP (the mature form of SREBP) is transported into the nucleus to induce the expression of genes involved in lipid synthesis and uptake. Conversely, excess cholesterol or oxysterol significantly inhibits the ER–Golgi trafficking of the SREBP–SCAP complex by promoting the association between SCAP and the ER‐resident INSIG protein, thereby blocking the expression of target genes.^44^, ^61^ Genetic studies have demonstrated that conditional knockout of SCAP or S1P in the liver effectively inhibits SREBP cleavage and SREBP‐mediated lipid synthesis,^101^, ^102^ indicating the therapeutic potential of SREBP inhibition in individuals with obesity, T2D, MAFLD, and atherosclerosis.

In 2011, Song and colleagues^103^ reported betulin (**4**), a triterpenoid mainly isolated from the bark of *Betula platyphylla* Suk., as a specific inhibitor of the SREBP pathway. Betulin significantly inhibited the generation of both n‐SREBP‐1 and n‐SREBP‐2 in sterol‐depleted CRL‐1601 rat hepatocytes. Furthermore, betulin was found to physically interact with SCAP, thereby promoting the association between SCAP and INSIG1 and contributing to its inhibition of SREBP maturation. However, the specific residues involved in the interaction between SCAP and betulin was still unknown. In addition, due to its specific inhibitory effect on SREBP processing, betulin potently downregulates SREBP target genes and reduces the de novo synthesis of both cholesterol and fatty acids, thereby decreasing cellular lipid levels in sterol‐depleted CRL‐1601 cells. Furthermore, betulin not only alleviated DIO, reduced lipid accumulation in serum and tissues, and increased insulin sensitivity in HFD‐fed mice but also reduced the size of atherosclerotic plaques in HFD‐fed LDL receptor (LDLR)‐knockout mice.

Although pharmacological activation of the LXR has protective effects against atherosclerosis, it also induces hepatic steatosis and hypertriglyceridemia by upregulating SREBP‐1c expression and inducing fatty acid synthesis.^104^ Unlike traditional SREBP inhibitors such as oxysterols, betulin does not affect LXR, thus exhibited higher safety.^103^ Overall, the specific inhibitory effect of betulin on the SREBP pathway without activating LXR ensures its suppressive effects on both cholesterol and fatty acid synthesis and thus potent therapeutic potential for metabolic disorders. Previously, betulin was found to inhibit the growth of several cancer cells.^105^ Since SREBPs signaling regulates lipid metabolism to meet the bioenergetics and biosynthetic demands of rapidly proliferating cancer cells.^106^ Therefore, betulin‐mediated inhibition of SREBPs signaling also contributed to its anticancer effects.

#### 3.2.2 Lycorine acts as a SCAP degrader that inhibits SREBP activity

Ideal SCAP inhibitors should also inhibit the SREBP pathway without activating ER stress, which has been observed with reported SCAP inhibitors such as betulin and fatostatin.^103^, ^107^ In 2020, Zheng et al.^108^ identified lycorine (**5**), a natural alkaloid isolated from *Lycoris radiata*, as a novel SCAP binder using Alpha Screen‐based CETSA. Furthermore, lycorine was found to interact with SCAP by forming two hydrogen bonds with Tyr793 and Ala1029, with a dissociation constant (*K*_D_) value of 15.2 ± 4.5 nM. Distinct from the results obtained with the other SCAP inhibitors, the direct interaction between lycorine and SCAP significantly decreased the protein level of SCAP in vitro at the posttranslational level without inducing ER stress or LXR transactivation in sterol‐depleted HL‐7702 cells. Therefore, lycorine can induce ubiquitin–proteasome‐mediated degradation of SREBPs, thereby inhibiting the maturation and transcriptional activity of SREBPs as well as decreasing the cellular levels of lipids.

By using different pathway inhibitors and genetic deletion strategies, lycorine treatment was found to dissociate SCAP from INSIG1 and SREBP1, increase SCAP protein export from the ER, and accelerate the degradation of SCAP in a lysosome‐dependent manner. Mechanistically, lycorine triggered the lysosomal translocation of SCAP by enhancing the interaction of SCAP with sequestosome 1 (SQSTM1), a cargo receptor and adaptor protein involved in autophagy and lysosomal degradation. Through promoting SCAP protein degradation, lycorine treatment significantly ameliorated HFD‐induced obesity, hyperlipidemia, hepatic steatosis, and insulin resistance in mice.^108^

#### 3.2.3 Corylin promotes mature SREBP degradation in an HSP90β‐dependent manner

The maturation and stability of SREBPs are strictly regulated by molecular chaperones, including heat shock protein 90 (HSP90).^109^ Although pan‐HSP90 inhibits proteins such as 17‐AAG and effectively enhances proteasome‐dependent degradation of SREBP, thereby resulting in the suppression of SREBP target genes and lipid biosynthesis in vitro and in vivo, pan‐HSP90 inhibitors have been shown to have severe side effects, including hepatotoxicity.^109^ Hence, developing selective inhibitors against HSP90 subtypes with decreased toxicity is an attractive strategy for the treatment of metabolic disorders. In 2019, Zheng et al.^110^ reported that HSP90β, rather than HSP90α, was overexpressed in the liver tissues of MAFLD patients and ob/ob mice. HSP90β knockdown significantly improved lipid homeostasis in HL‐7702 hepatocytes and HFD‐induced obese mice.^110^ Therefore, HSP90β represents a potential therapeutic target for lipid disorders.

By using virtual screening and a luciferase reporter gene, corylin (**6**), an isoflavone found mainly in the fruits of *Psoralea corylifolia* Linn., was identified as a new selective HSP90β inhibitor and inhibitor of the SREBP pathway.^110^ Corylin was found to selectively bind to the middle domain of HSP90β with a *K*_D_ of 24.7 ± 10.2 nM by forming hydrogen bonds with Trp312, Asn375, and Asn436. Interestingly, corylin did not directly inhibit the binding of HSP90β to SREBPs but disrupted the interaction between HSP90β and Akt and significantly reduced Thr308 phosphorylation of Akt and Ser9 phosphorylation of GSK3β, thereby promoting F‐Box‐ and WD40 domain protein‐7‐mediated ubiquitination and proteasomal degradation of mature SREBPs.

In preclinical models, corylin treatment effectively improved Western‐type diet‐induced dyslipidemia, insulin resistance, and atherosclerosis in mice by inhibiting HSP90β.^110^ Like the SCAP binder betulin and the SCAP degrader lycorine, corylin did not activate the expression of LXR target genes or ER stress genes,^110^ which may confer improved safety in the treatment of chronic metabolic disorders.

#### 3.2.4 Artepillin C disrupts the interaction between CREB and CRTC2 to inhibit gluconeogenesis and lipogenesis

T2D patients exhibit abnormal gluconeogenesis in the liver, which results in fasting hyperglycemia and insulin resistance.^28^, ^29^ Pathophysiologically, excessive glucagon not only causes activation of cAMP–PKA–CREB signaling and CREB nuclear localization but also induces the dephosphorylation and nuclear translocation of CRTC2. In the nucleus, CREB interacts with CRTC2, thereby inducing the transcription of gluconeogenic genes, such as PGC1α, phosphoenolpyruvate carboxy kinase, and glucose‐6‐phosphatase.^29^ The inhibition of CREB or CRTC2 has been proven to repress gluconeogenesis in mice and may be a potential therapeutic strategy for treating metabolic disorders.^111^

In 2022, Chen and colleagues^112^ identified artepillin C (**7**), a phenolic acid extracted from Brazilian green propolis, as a potent inhibitor of the CREB–CRTC2 interaction using a mammalian two‐hybrid assay, the results of which showed an IC_50_ value of 24.5 ± 0.5 µM. Furthermore, SPR and pull‐down analyses confirmed that artepillin C could directly bind to CREB, thereby disrupting the formation of the CREB–CRTC2 complex. However, the binding sites of artepillin C on CREB are still unknown. Moreover, artepillin C did not obviously alter CREB phosphorylation or nuclear translocation of CRTC2 but did significantly decrease the expression of key gluconeogenic genes in primary hepatocytes. After oral administration, artepillin C was mainly enriched in liver tissue and significantly improved obesity and insulin resistance in DIO mice and db/db mice but did not cause obvious toxicity in lean mice.

In addition to gluconeogenesis, the CREB–CRTC2 complex is also implicated in lipid metabolism.^113^ Artepillin C was found to decrease the mRNA and mature protein form of SREBP1/2 by blocking the CREB/CRTC2–cAMP‐response element (CRE)–LXRα axis, therefore markedly reducing the expression of SREPB target genes involved in cholesterol synthesis, cholesterol uptake, and fatty acid synthesis in the livers of DIO mice.^112^ Therefore, artepillin C also exhibited potent antihyperlipidemic effects on DIO and db/db mice by blocking SREBP‐mediated lipid synthesis. Taken together, these findings suggest that artepillin C effectively alleviates insulin resistance and glycolipid disorders by blocking the CREB–CRTC2 interaction and CREB–CRTC2/SREBP‐mediated gene transcription in gluconeogenesis and lipogenesis. These data also indicate that CREB/CRTC2 is an ideal target for developing antimetabolic disorders drugs.

### 3.3 Natural products that promote lipid breakdown and energy consumption

**FIGURE 6:**
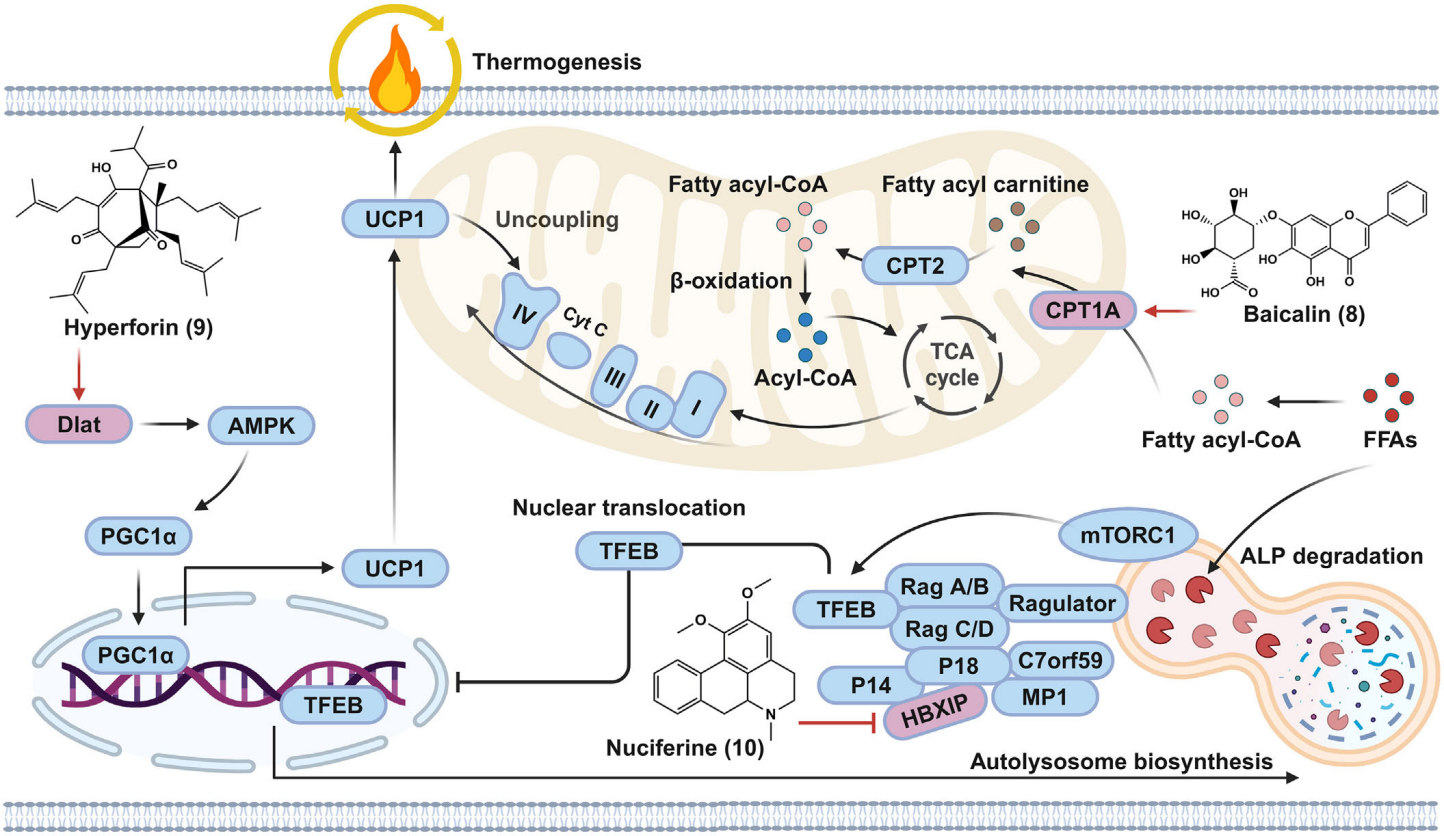
The functional targets and underlying mechanisms of natural small molecules that promote lipid consumption and elimination. Baicalin (**8**) acts as a CPT1A activator to promote FAO‐mediated lipid consumption. Hyperforin (**9**) binds to Dlat, thereby activating Dlat–AMPK–PGC1α–UCP1 axis to enhance thermogenesis. Nuciferine (**10**) directly interacts with HBXIP, thereby activating TFEB‐mediated ALP. C7orf59, Ragulator complex protein LAMTOR4; CoA, coenzyme A; Cyt *C*, cytochrome *C*; MP1, MEK‐binding partner 1; P14, endosomal adaptor protein p14; P18, lipid raft adaptor protein p18; Rag A/B/C/D, Ras‐related GTP‐binding protein A/B/C/D; TCA cycle, tricarboxylic acid cycle.

As shown in Figure 6, we summarized the molecular mechanisms and functional targets of natural small molecules on the improvement of metabolic disorders via enhancing lipid breakdown and energy consumption. For instance, baicalin (**8**) targeted CPT1A, which is the rate‐limiting enzyme of FAO. Hyperforin (**9**) exhibited thermogenic effect via activating AMPK–PGC1α–UCP1 axis in a dihydrolipoamide S‐acetyltransferase (Dlat)‐dependent manner. Nuciferine (**10**) acted as a suppressor of hepatitis B X‐interacting protein (HBXIP), which is involved in the inhibition of ALP.

#### 3.3.1 Baicalin allosterically activates hepatic CPT1 to ameliorate DIO and hepatic steatosis

In addition to inhibiting lipogenesis by regulating the SREBP pathway, it is also possible to inhibit lipid accumulation and ameliorate metabolic disorders by increasing lipid expenditure.^114^ Mitochondrial FAO, an enzymatic biochemical process that converts FFAs into acetyl‐CoA, is the major pathway of lipid consumption.^115^ A series of enzymes and transporters involved in FAO may be targeted to promote FAO and inhibit lipid accumulation.

Several natural flavonoids have been reported to ameliorate lipid disorders mainly by regulating lipogenic pathways.^116^ Among them, baicalin (**8**), a major active component of *Scutellaria baicalensis*, was found to attenuate HFD‐induced hepatic steatosis in rodents, but the underlying mechanism and cellular target of baicalin remain unknown.^117^ Da et al.^118^ discovered that baicalin directly bound to carnitine palmitoyltransferase 1A (CPT1A), a rate‐limiting enzyme of FAO. Interestingly, baicalin significantly increased the enzymatic activity of CPT1A, thereby contributing to its suppressive effect on high fatty acid (HFA)‐induced lipid accumulation in cells. Molecular docking further demonstrated that baicalin bound to several residues, including Lys286, Ile291, Glu309, and His327, in a predicted allosteric binding pocket, and these amino acids were essential for CPT1A activation by baicalin. Through activating FAO‐mediated energy expenditure in mice, baicalin ameliorated DIO and associated metabolic disorders, such as insulin resistance, hyperlipidemia, hyperglycemia, and hepatic steatosis. Furthermore, the antiobesity and antisteatotic effects of baicalin were almost completely abolished by hepatic CPT1A knockdown, which could be rescued by overexpressing wild‐type CPT1A but not the baicalin‐insensitive His327Glu mutant in liver tissues.^118^ Previously, the extract of *S. baicalensis* has been confirmed to exhibit a strong lipid‐reducing effect,^119^ which may be attributed to its main active ingredient baicalin‐mediated activation of CPT1A.

Collectively, by directly binding to CPT1A and allosterically activating its activity to accelerate fatty acid oxidation, baicalin effectively ameliorated DIO, hepatic steatosis, and metabolic disorders.^118^ Because of its high safety, baicalin thus can act as a candidate for the treatment of metabolic disorders. Additionally, several flavonoids, such as baicalein and scutellarein, possess the same skeleton as baicalin. However, further studies are needed to determine whether these baicalin analogs also activate the CPT1A enzyme in a similar manner and to determine their structure–activity relationships, which could guide the design of selective CPT1A activators with increased activity and improved safety.

#### 3.3.2 Hyperforin alleviates obesity by binding to Dlat and activating the AMPK–PGC1α–UCP1 axis

In mammals, adipose tissues, including brown adipose tissue (BAT), beige adipose tissue, and WAT, play critical roles in the homeostasis of body temperature and energy metabolism. Among these, WAT is mainly responsible for energy storage and can also transdifferentiate into beige adipocytes (brown phenotype), a process called browning.^120^ BAT and beige adipocytes can convert energy into heat through UCP1‐mediated thermogenesis.^121^ In addition to promoting the FAO pathway, enhancing WAT browning and/or upregulating BAT thermogenesis are considered other effective strategies for treating obesity and metabolic disorders.^122^

Because cold exposure can directly stimulate WAT browning and BAT thermogenesis, Liu and collaborators^123^ used the Connectivity Map approach to identify hyperforin (**9**), an active ingredient of *Hypericum perforatum* (St John's wort), as a candidate mimetic of the “signatures” of cold‐induced gene expression in WAT and BAT. In C3H10T1/2‐derived adipocytes, primary inguinal adipocytes differentiated from the stromal vascular fraction (SVF) of mouse inguinal WAT and from human mesenchymal stem cell‐derived adipocytes, and hyperforin treatment produced smaller lipid droplets and higher thermogenic gene levels (such as those of PGC1α and UCP1), elevated the number of mitochondria, and enhanced cellular respiration. Furthermore, hyperforin also promoted thermogenesis to a similar extent in primary brown adipocytes derived from mouse SVFs. In ob/ob mice and DIO mice, hyperforin substantially decreased the weight of inguinal WAT but not the weight of lean mass, epididymal WAT or BAT. Furthermore, the antiobesity effects of hyperforin were associated with its facilitation of thermogenesis via inguinal WAT browning and BAT activation. Mechanistically, hyperforin‐mediated thermogenesis was dependent on activation of the AMPK–PGC1α–UCP1 signaling axis.

By applying limited proteolysis‐mass spectrometry (LiP‐SMap) combined with microscale thermophoresis (MST) analysis and molecular docking, the authors confirmed that Dlat is a direct cellular target of hyperforin. Upon binding to the C‐terminal inner domain of Dlat via interactions with Ser516, Arg549, and Asn567, hyperforin effectively upregulated Dlat protein abundance and AMPK phosphorylation, thereby promoting UCP1 expression as well as adipocyte browning and thermogenesis in vitro and in vivo.^123^ Therefore, these findings not only suggest that hyperforin is a promising lead compound for obesity therapy through activation of the Dlat–AMPK–PGC1α–UCP1 axis and promotion of energy expenditure, but also propose that Dlat acts as an attractive target for developing antiobesity drugs.^123^ However, the molecular mechanism of Dlat‐mediated AMPK activation requires further characterization.

#### 3.3.3 Nuciferine interacts with HBXIP to activate the TFEB‐mediated ALP

In addition to clearing misfolded proteins and damaged organelles, mTORC1–TFEB‐mediated ALP can also induce the degradation of macromolecules and lipid droplets, thereby resulting in their breakdown into essential metabolic intermediates, including glucose, amino acids, and FFAs.^59^, ^60^ However, sustained nutritional overload causes mTORC1 hyperactivation and impairment of TFEB‐mediated ALP, thereby leading to lipid accumulation and MAFLD.^72^ Therefore, reactivation of ALP via regulation of the mTORC1–TFEB axis has great therapeutic potential in MAFLD treatment.^124^

Nuciferine (**10**), an active alkaloid extracted from lotus leaves, has been shown to exhibit favorable results against hepatic steatosis, but the underlying mechanism involved remains unknown.^125^ Using RNA sequencing and enrichment analysis, Du et al.^126^ reported that ALP induction was involved in the therapeutic benefits of nuciferine on HFD‐induced MAFLD mice and PA‐stimulated hepatocytes. Further study indicated that nuciferine effectively enhanced autophagic flux by increasing SQSTM1 degradation and formation of phosphotidylethanolamine conjugated form of microtubule associated protein 1 light chain 3 (LC3‐II), and promoted lysosome biogenesis by increasing the protein abundance of lysosomal‐associated membrane protein 1 (LAMP1) and cathepsin D and the activity of lysosomal proteases.^126^

The nuciferine‐mediated activation of ALP and improvement in steatosis and insulin resistance were mainly associated with the activation of TFEB. CETSA and  co‐immunoprecipitation revealed that nuciferine directly binds to the Ragulator subunit HBXIP) and impairs the interaction between the Ragulator complex and Rag GTPases,^126^ which is crucial for the activation of mTORC1.^127^ Due to its interaction with HBXIP and inhibition of mTORC1, nuciferine could activate TFEB‐mediated ALP by inhibiting TFEB phosphorylation at Ser211. Taken together, these results suggested that nuciferine may be a promising candidate for MAFLD treatment and that modulation of the mTORC1–TFEB–ALP axis represents a novel pharmacological therapy for MAFLD. In addition to nuciferine, several natural products, such as HEP‐14/15 and naringenin, have been identified as ALP activators that activate TFEB‐mediated signaling,^128^, ^129^ but whether these compounds exert protective effects on MAFLD remains to be studied.

### 3.4 Natural products that block metabolic inflammation

**FIGURE 7:**
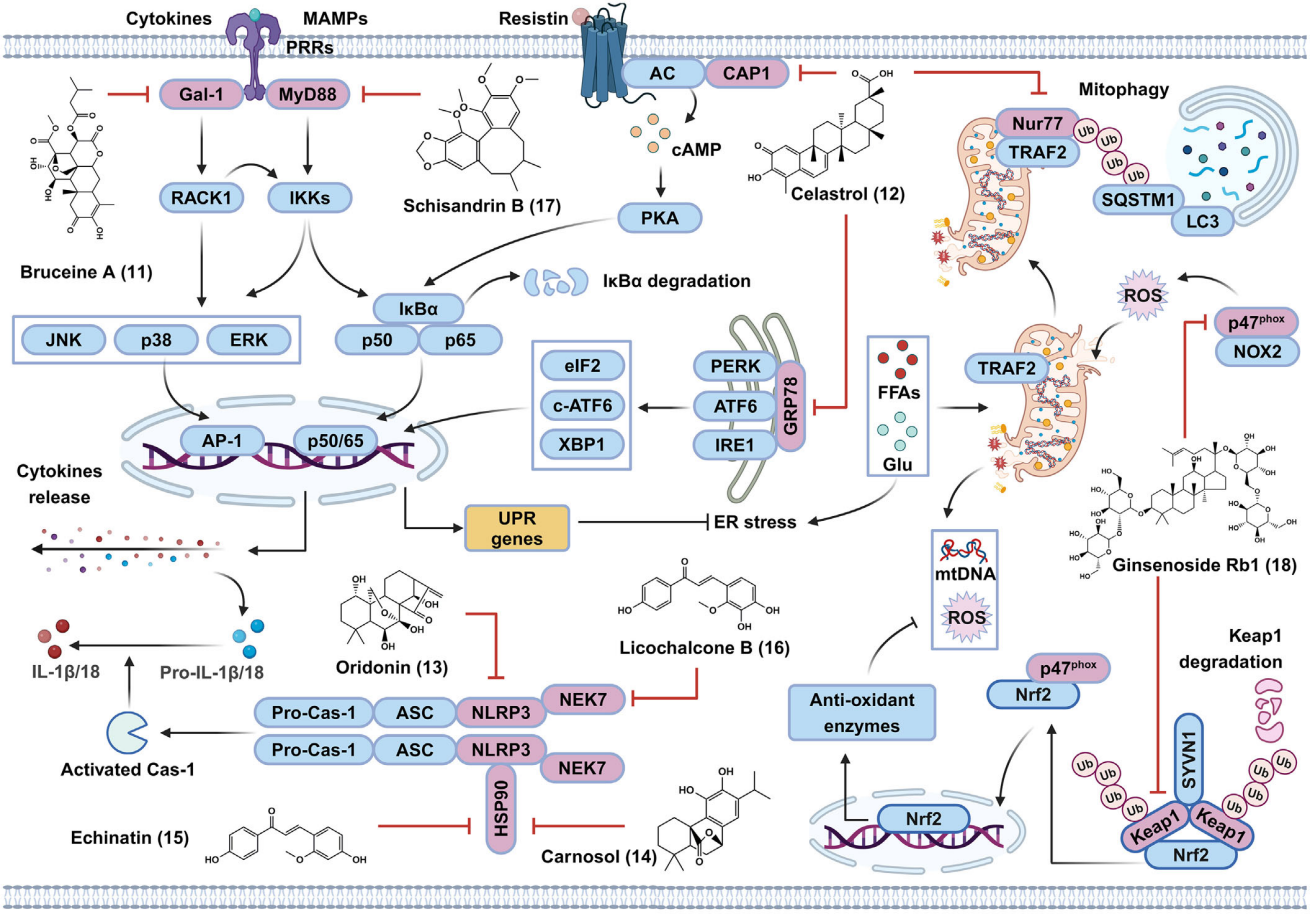
The functional targets and underlying mechanisms of natural bioactive components against metabolic inflammation and oxidative stress. Bruceine A (**11**) and schisandrin B (**17**) binds to Gal‐1 or MyD88 respectively, thereby inhibiting the activation of NF‐κB and MAPK as well as metabolic inflammation. Celastrol (**12**) directly interacts with CAP1, Nur77, and GRP78, thereby blocking metabolic inflammation‐associated NF‐κB activation, activating mitophagy, and relieve ER stress, respectively. Furthermore, oridonin (**13**), carnosol (**14**), echinatin (**15**), licochalcone B (**16**) can inhibit NLRP3 inflammasome activation via directly binding to NLRP3, HSP90, and NEK7. In addition, ginsenoside Rb1 (**18**) can interact with Keap1 and p47^phox^, thereby activating Nrf2 antioxidant pathway and inhibiting NOX2‐mediated ROS generation, respectively. AC, adenylate cyclase; AP‐1, activator protien‐1; c‐ATF6, cleaved ATF6; eIF2, eukaryotic translation initiation factor 2; p50/p65, NF‐κB p50/p65 subunits; two UPR, unfolded protein response; XBP1, X‐box‐binding protein 1.

In this section, the anti‐inflammatory targets and metabolic benefits of natural bioactive components were reviewed in Figure 7. Among them, bruceine A (**11**) was found to target galectin‐1 (Gal‐1), a PRR implicated in the activation of NF‐κB and MAPK pathways. The inhibitory effects of celastrol (**12**) on metabolic inflammatory were associated with its direct cellular targets, including resistin's receptor adenylyl cyclase‐associated protein 1 (CAP1), orphan nuclear receptor Nur77, ER stress marker glucose‐regulated protein (GRP78), respectively. Furthermore, oridonin (**13**) was able to directly bind to NLRP3; carnosol (**14**) and echinatin (**15**) inhibited NLRP3 inflammasome via targeting the chaperon HSP90; licochalcone B (**16**) inhibited NLRP3 inflammasome by binding to the inflammasome component NEK7. In addition, schisandrin B (**17**) targeted MyD88, an adaptor involved in the activation of NF‐κB and MAPK.

#### 3.4.1 Bruceine A directly interacts with Gal‐1 to alleviate diabetic kidney disease

Upon metabolic overload, metabolism‐related danger signaling molecules, such as ox‐LDL and AGEs, can activate a series of proinflammatory signaling cascades, such as NF‐κB and MAPK cascades, through binding to several cell‐surface receptors. The activation of these pathways further promotes the release of proinflammatory cytokines and chemokines, thereby contributing to the initiation and progression of insulin resistance and metabolic disorders.^63^, ^64^ Therefore, blocking metabolic overload‐induced proinflammatory pathways by targeting critical signaling molecules may be a promising strategy for the treatment of metabolic disorders.^130^

In 2022, Li et al.^131^ reported that bruceine A (**11**), a natural diterpenoid extracted from the fruit of *Brucea javanica*, exhibited potent renoprotective effects on high glucose (HG)‐stimulated rat mesangial HBZY‐1 cells and db/db mice through the inhibition of the NF‐κB, JNK/p38 MAPK, and Akt pathways as well as the production of cytokines and chemokines. By using a biotinylated bruceine A probe, Gal‐1, a member of the galectin family that functions as a PRR involved in many chronic inflammatory diseases including diabetic nephropathy, was identified as the specific cellular target of bruceine A. Furthermore, the authors demonstrated that bruceine A could directly bind to recombinant human Gal‐1 with a *K*_D_ value of 9.0 µM by forming hydrogen bonds with His44 and Arg48. By selectively binding to Gal‐1, bruceine A was able to disturb the interaction between Gal‐1 and the receptor for activated protein C kinase 1 (RACK1), thereby suppressing Gal‐1‐mediated activation of the NF‐κB, JNK/p38 MAPK, and Akt pathways in HG‐stimulated HBZY‐1 cells. As such, bruceine A is a novel Gal‐1 inhibitor that possesses robust anti‐inflammatory properties and protects against diabetic nephropathy.^131^ This study may also lead to the use of new pharmacological therapeutics for diabetic nephropathy and other inflammation‐associated metabolic disorders.

#### 3.4.2 Celastrol inhibits metabolic inflammation in a multitarget manner

Celastrol (**12**), a quinone methide triterpenoid isolated from the root of *Tripterygium wilfordii*, has a range of pharmacological effects, such as anticancer, anti‐inflammatory, and neuroprotective effects.^132^ In 2015, Ozcan's group^133^ reported that celastrol effectively reduced HFD‐induced body weight gain by activating the hypothalamic leptin receptor‐signal transducer and activator of transcription 3 (STAT3) pathway in DIO mice but not ob/ob or db/db mice. In 2019, they further demonstrated that IL‐1 receptor 1 was required for the leptin sensitization and antiobesity effects of celastrol.^134^ These findings strongly suggest that celastrol has an anti‐inflammatory role in antiobesity effects, but the functional target proteins of celastrol are unclear.

Resistin, a cytokine produced by adipose tissue, has been found to cause metabolic inflammation by activating the NF‐κB pathway and promoting proinflammatory gene expression, thereby resulting in insulin resistance in adipose and liver tissues.^135^ Thus, resistin signaling acts as a bridge connecting inflammation and metabolic disorders and represents a valuable target for the treatment of metabolic diseases.^135^ In 2021, through target‐responsive accessibility profiling, Sun's laboratory demonstrated that adenylyl CAP1, a functional receptor of resistin, was the direct cellular target of celastrol in macrophages.^136^ Mechanistically, the binding of celastrol to CAP1 disrupted the interaction between CAP1 and resistin, thereby reducing the resistin‐induced elevation of intracellular cAMP, inhibiting cAMP‐mediated activation of the PKA/NF‐κB pathway and the production of proinflammatory cytokines in THP1 cells. In DIO mice, celastrol significantly reduced body weight, enhanced insulin sensitivity, and inhibited hepatic steatosis via the inhibition of resistin‐mediated inflammation in adipose and liver tissues.^136^

Furthermore, Zhang et al.^137^ identified celastrol as a potent binder of the nuclear hormone receptor NUR/77 (Nur77), with a *K*_D_ of 292 nM. Nur77 is an orphan nuclear receptor that participates in the regulation of NF‐κB activation and energy homeostasis.^138^, ^139^ Accordingly, celastrol exerted significant anti‐inflammatory and antiobesity effects on TNF‐α‐stimulated HepG2 cells and DIO mice in a Nur77‐dependent manner. Mechanistically, celastrol binding induced Nur77 translocation to mitochondria, where Nur77 bound to the E3 ubiquitin ligase TNF receptor‐associated factor 2 (TRAF2) via the interaction between the ligand‐binding domain of Nur77 and the LxxLL motif of TRAF2. Meanwhile, the celastrol‐induced interaction between Nur77 and TRAF2 inhibited TNF‐α‐induced and TRAF2‐mediated receptor‐interacting protein kinase 1 ubiquitination and activation of downstream IKK–NF‐κB signaling. Furthermore, the celastrol‐induced interaction between Nur77 and TRAF2 also resulted in TRAF2‐mediated Lys63‐linked ubiquitination of Nur77. Ubiquitinated Nur77 then interacts with SQSTM1 in mitochondria, thereby leading to mitophagy in damaged mitochondria and mitigation of metabolic inflammation.^137^ This study suggested that clearing damaged mitochondria via mitophagy represents a novel strategy against metabolic inflammation.

Additionally, continuous metabolic stress also disrupts ER homeostasis and causes ER stress in parenchymal and immune cells, resulting in insulin resistance and inflammation, ultimately triggering or exacerbating metabolic disorders.^140^ Excess metabolites in the ER compartment can disrupt the interactions of 78 kDa GRP78 with inositol‐requiring enzyme 1, protein kinase R‐like ER kinase, and activating transcription factor 6 (ATF6), leading to ER stress and metabolic inflammation.^141^ Previously, celastrol was shown to reduce ER stress; however, the exact molecular mechanisms and target proteins involved have not been determined.^142^ In 2022, Rong's group^143^ showed that GRP78 was the major celastrol‐bound protein in RAW264.7 murine macrophages. Celastrol covalently bound to the Cys41 residue of GRP78 via a Michael addition reaction, thereby blocking the chaperone activity of GRP78, and reducing the expression of ER stress biomarkers in PA‐stimulated RAW264.7 cells. Moreover, due to its inhibition of ER stress in the liver and epididymal adipose tissue, celastrol effectively reduced body weight, suppressed inflammation and lipogenesis while promoting hepatic lipolysis in DIO mice.^143^ Therefore, covalent inhibition of GRP78 represents a novel antiobesity mechanism for reprogramming the signaling networks involved in ER stress, inflammation, and lipid metabolism in liver and adipose tissues.

Metabolic inflammation can upregulate the expression of hypothalamic PTP1B, a major negative regulator of insulin and leptin signaling via dephosphorylation of the JAK2–STAT3 axis.^144^ Interestingly, celastrol was identified as a potent PTP1B inhibitor.^145^ Therefore, the leptin‐sensitizing and antiobesity effects of celastrol were partially attributed to its inhibition of PTP1B enzymatic activity. Moreover, celastrol was identified as an HSP90 inhibitor.^146^ As HSP90 is critical for proinflammatory signaling transduction and SREBP maturation,^109^ the antiobesity, anti‐inflammatory, and leptin sensitization effects of celastrol were associated, at least in part, with its HSP90 inhibitory activity. In addition, celastrol has also been proven to reduce HFD‐induced obesity through enhancing heat shock factor 1 (HSF1)–PGC1α axis‐mediated energy expenditure.^147^ Coincidentally, celastrol increased HSF1 protein abundance and triggered a heat shock response by inhibiting HSP90–CDC37 interaction.^146^ Therefore, celastrol‐mediated energy expenditure may also be attributed to its ability to inhibit HSP90. Taken together, these findings indicate that celastrol may be a promising candidate for the treatment of metabolic disorders in a multitarget manner and that celastrol thus can be used as a lead agent for developing new therapies to treat metabolic disorders.

#### 3.4.3 Inhibition of NLRP3 by oridonin or other natural products to alleviate diet‐induced metabolic disorders

Sustained metabolic stress induces the accumulation of metabolites, including cholesterol crystals, AGEs, and ox‐LDL, and causes the release of mtDNA, mtROS, and adenosine triphosphate (ATP) from damaged cells, which further induces the assembly and activation of the NLRP3 inflammasome, thereby leading to the maturation and release of IL‐1β and IL‐18 and aggravating metabolic disorders.^65^, ^70^ Hence, the NLRP3 inflammasome forms a nexus linking inflammation and metabolic disorders during metabolic stress, which provides new avenues for the treatment of metabolic disorders.^65^, ^70^

In 2018, Zhou's group identified oridonin (**13**), the major bioactive ent‐kaurane diterpenoid found in *Rabdosia rubescens*, as a specific NLRP3 inflammasome inhibitor.^148^ In murine bone marrow‐derived macrophages (BMDMs), oridonin significantly blocked lipopolysaccharide (LPS)/nigericin‐, monosodium urate crystal‐, or ATP‐induced NLRP3 inflammasome activation. Furthermore, LPS‐induced caspase‐1 activation and IL‐1β secretion in human peripheral blood mononuclear cells could also be suppressed by oridonin. Subsequently, oridonin was able to disrupt the interaction between NLRP3 and NIMA‐related kinase 7 (NEK7), which is essential for the assembly and activation of the NLRP3 inflammasome. By using a biotinylated oridonin probe, oridonin was found to interact with NLRP3 by forming a covalent bond between the α,β‐unsaturated carbonyl moiety of oridonin and Cys279 located in the nucleotide‐binding and oligomerization domain of NLRP3, thereby blocking the NLRP3–NEK7 interaction and subsequent inflammasome assembly and activation. In addition, oridonin treatment also exhibited potent therapeutic effects on HFD‐induced body weight gain, hyperglycemia, hyperlipidemia, insulin resistance, and metabolic inflammation, which were obviously abolished by NLRP3 knockout in mice.^148^ These results suggested that oridonin specifically inhibited the activation of the NLRP3 inflammasome to treat metabolic disorders.

Activation of the NLRP3 inflammasome is also controlled by a series of posttranscriptional mechanisms,^149^ which represents a new approach for inhibiting NLRP3 activation. In 2020 and 2021, Xiao's group^150^, ^151^ successively identified two new NLRP3 inhibitors, carnosol (the main active ingredient of rosemary and sage) and echinatin (an active chalcone mainly found in licorice). Unlike oridonin, carnosol (**14**)^150^ and echinatin (**15**)^151^ could directly bind to HSP90, which is essential for NLRP3 inflammasome activity.^152^ Upon binding to HSP90, carnosol and echinatin both inhibited the ATPase activity of HSP90, thereby disrupting the association between NLRP3 and HSP90 and inhibiting subsequent NLRP3 inflammasome assembly and activation. Furthermore, treatment with carnosol or echinatin effectively protected mice from methionine‐ and choline‐deficient (MCD) diet‐induced NASH by inhibiting NLRP3 inflammasome‐associated inflammation.^150^, ^151^

In addition to binding to NLRP3 or HSP90, targeting NEK7 is another alternative strategy for inhibiting the NLRP3 inflammasome.^153^ In 2022, Xiao's group^154^ demonstrated that licochalcone B (**16**), a main component of licorice, was a specific inhibitor of the NLRP3 inflammasome. They further identified NEK7 as the functional target of licochalcone B in BMDMs. Upon interacting with NEK7, licochalcone B did not affect the kinase activity of NEK7 but obviously inhibited the NLRP3‐NEK7 interaction, thus repressing NLRP3 inflammasome assembly and activation. In vivo, licochalcone B was found to alleviate MCD diet‐induced NASH and metabolic inflammation.^154^ Furthermore, berberine, a main active ingredient of *Coptis chinensis*, was also identified as a binder of NEK7 in 2021.^155^ Unlike licochalcone B, berberine potently inhibited the ATPase activity of NEK7, with an IC_50_ of 4.2 µM, by forming a hydrogen bond with Arg121, thereby markedly blocking the NLRP3 inflammasome activation.^155^ Previously, berberine was shown to mitigate metabolic disorders through inhibiting gluconeogenesis, reducing LDL content, and enhancing insulin sensitivity.^156^ However, further validation is needed to determine whether the improvement effect of berberine on metabolic disorders depends on the NEK7–NLRP3 pathway. Overall, these findings further support the concept of NLRP3 as a therapeutic target for metabolic disorders. Moreover, these natural products are expected to become candidates for treating NLRP3‐driven diseases, including metabolic disorders.

#### 3.4.4 Schisandrin B attenuates diabetic cardiomyopathy by targeting MyD88

Metabolic overload‐derived danger molecules, including AGEs and ox‐LDL, can be sensed by multiple PRRs to induce proinflammatory pathways and metabolic inflammation.^63^ In 2020, Liang's group reported that AGEs could directly bind to myeloid differentiation factor 2 (MD2), the coreceptor of TLR4. Upon AGE binding to the MD2–TLR4 complex, MyD88 and Toll/interleukin‐1 receptor (TIR) domain‐containing adapter‐inducing interferon‐β (TRIF) are recruited to the AGE–MD2–TLR4 complex, resulting in the activation of downstream signaling pathways to produce proinflammatory cytokines and mediators.^157^ This evidence provides new mechanistic insight linking HG, AGEs, and MD2–TLR4–MyD88/TRIF signaling in metabolic disorders, and inhibition of the critical proteins in this cascade may confer therapeutic efficacy against metabolic disorders.

In 2022, Liang's group further reported that schisandrin B (**17**), a bioactive dibenzooctadiene lignan enriched in *Schisandra chinensis*, markedly mitigated HG‐induced hypertrophic and fibrotic responses in cardiomyocytes.^158^ Furthermore, RNA sequencing and pathway enrichment analyses revealed that the inflammatory response was involved in the protective effect of schisandrin B on HG‐stimulated cardiomyocytes. Moreover, schisandrin B was found to effectively block the HG‐induced expression of MyD88‐dependent genes (such as TNF‐α and IL‐6) through inhibiting the TLR4–MyD88 interaction and downstream transforming growth factor‐beta‐activated kinase 1‐mediated activation of the NF‐κB and MAPK pathways but not the TRIF‐dependent activation of the TRAF‐associated NF‐κB activator‐binding kinase 1‐interferon regulatory factor 3 signaling axis. The authors further demonstrated that schisandrin B was able to bind to MyD88 by interacting with Thr272 and Arg288 in the TIR domain, thereby inhibiting the TLR4–MyD88 interaction and MyD88‐dependent proinflammatory pathways. Furthermore, schisandrin B treatment obviously attenuated cardiac hypertrophy and fibrosis as well as MyD88‐dependent inflammatory responses in db/db mice and STZ‐induced type 1 diabetic mice.^158^ These data suggested the protective effects of schisandrin B against diabetic cardiomyopathy via the specific targeting of MyD88 and downstream proinflammatory pathways. In 2018−2021, Liang's group also identified several natural MD2 inhibitors, such as baicalein, cardamonin, and shikonin,^159^, ^160^, ^161^ which have also been demonstrated to have therapeutic potential for metabolic disorders.^162^, ^163^, ^164^ The therapeutic effects of these compounds against metabolic disorders, at least in part, were attributed to the suppression of MD2‐mediated activation of proinflammatory signaling cascades.

### 3.5 Natural products that ameliorate metabolic disorders through other molecular mechanisms

Herein, we reviewed two natural bioactive compounds exhibiting protective effects against metabolic disorders via inhibiting oxidative stress and regulating circadian rhythm, respectively. Ginsenoside Rb1 (**18**; Figure 7) not only targeted Keap1, the suppressor of Nrf2 antioxidant pathway, but also bound to p47^phox^, a subunit of activated NOX2. Nobiletin (**19**) acted as an agonist for the ROR nuclear receptors in the circadian oscillator.

#### 3.5.1 Ginsenoside Rb1 attenuates diabetic atherosclerosis by reducing oxidative stress in a Keap1/p47^phox^‐dependent manner

Metabolic overload, such as hyperglycemia, causes ROS overproduction by activating NOX‐mediated metabolic pathways.^76^, ^77^ Excess ROS induce endothelial dysfunction, which is a central player in the pathogenesis of metabolic disorders and complications, including diabetic atherosclerosis.^165^ Conversely, the Nrf2 pathway can neutralize ROS by inducing the expression of several antioxidant genes, including heme oxygenase 1 (HMOX1). Pharmacological activation of Nrf2 signaling and/or inhibition of NOXs may constitute a promising strategy for attenuating ROS‐mediated endothelial dysfunction and diabetic atherosclerosis.^166^

In 2022, Wang et al.^167^ identified ginsenoside Rb1 (**18**), a main active ingredient of ginseng, as a dual Kelch‐like ECH‐associated protein 1 (Keap1) and 47 kDa component of the phagocyte NADPH oxidase (p47^phox^) inhibitor in endothelial cells (ECs) using a luciferase reporter assay, SPR, and MST. Keap1 functions as an adaptor to recruit Nrf2 to the Keap1–Cullin3 E3 ligase complex for ubiquitination, which causes Nrf2 degradation.^168^ Herein, the binding of Rb1 to Keap1 significantly promoted the ubiquitination of Keap1 at Lys108, Lys323, and Lys551 through the recruitment of the E3 ligase synovial apoptosis inhibitor 1 (SYVN1), thereby leading to the proteasomal degradation of Keap1. Rb1‐mediated Keap1 degradation further resulted in the nuclear translocation of Nrf2, the formation of the Nrf2–PGC1α complex, and the induction of HMOX1 in ox‐LDL/HG‐stimulated EA.hy926 ECs.^167^

The p47^phox^ protein is a subunit of activated NOX2 that generates ROS in ECs.^169^ Upon binding to p47^phox^, Rb1 potently decreases the protein abundance, phosphorylation, and membrane translocation of p47^phox^, thereby blocking the assembly of the NOX2 complex. Interestingly, Rb1 effectively promoted the dissociation of p47^phox^ from the Keap1–p47^phox^ complex and enhanced the interaction between p47^phox^ and Nrf2.^167^ As the p47^phox^–Nrf2 interaction contributes to the nuclear accumulation of Nrf2,^170^ Rb1 also activates the Nrf2 pathway by promoting the p47^phox^–Nrf2 interaction. Due to its activation of Nrf2 and inhibition of NOX2, Rb1 not only inhibited ox‐LDL/HG‐induced mitochondrial injury, ROS production, and inflammation in ECs, but also effectively attenuated aortic atherosclerotic plaque formation and reduced oxidative stress and inflammation in STZ/HFD‐challenged ApoE^−/−^ mice.^167^ Taken together, these findings suggest that Rb1 simultaneously interacts with Keap1 and p47^phox^ to activate Nrf2 and inhibit NOX2, thereby achieving the goal (inhibition of oxidative stress) of killing two birds (Keap1 and p47^phox^) with one stone (Rb1). Therefore, Rb1 represents a valuable candidate for the treatment of diabetic atherosclerosis or oxidative stress‐associated disorders.

#### 3.5.2 Nobiletin targets ROR receptors to enhance circadian rhythms

In mammals, the circadian locomotor output cycles kaput (CLOCK)/basic helix‐loop‐helix ARNT‐like protein 1 (BMAL1)‐ and neuronal Per‐Arnt‐Sim domain‐containing protein 2 (NPAS2)/BMAL1‐mediated transcriptional activation pathway and period1/2‐mediated autorepression pathway drive the rhythmic expression of downstream target genes involved in various metabolic processes, including glucose metabolism, lipid homeostasis, and thermogenesis.^87^ Recently, circadian rhythm dysfunction has been demonstrated to cause metabolic disorders, such as obesity, hyperglycemia, and hyperlipidemia.^88^ Although adjusting the feeding rhythm has been shown to have valuable therapeutic potential for metabolic disorders, its low compliance justifies the development of small molecule therapies that regulate circadian rhythms.

In 2016, by using heterozygous Clock^Δ19/+^ PER2::Luc reporter cells, which exhibit sustained reporter rhythms with a damped amplitude relative to that of wild‐type cells, He et al.^171^ reported that nobiletin (**19**), a polymethoxyflavone enriched in Citrus fruits, significantly enhanced the amplitude of circadian rhythms. Interestingly, the enhancement of clock function was observed in peripheral tissue but not in the brain. Furthermore, nobiletin treatment obviously reduced body weight, increased energy expenditure, enhanced glucose tolerance and insulin sensitivity, and alleviated liver lipid accumulation and steatosis in DIO mice in a Clock‐dependent manner. Moreover, similar protective effects of nobiletin against metabolic disorders were also observed in db/db mice but not in db/db or Clock^Δ19/Δ19^ double‐mutant mice. Furthermore, nobiletin largely restored clock rhythm proteins (e.g., BMAL1) and regulated clock‐controlled metabolic output genes (e.g., the lipid transferase CIDEC). Moreover, nobiletin was found to bind to the ligand binding domain of retinoid‐related orphan receptor‐alpha/gamma (RORα/γ), thereby enhancing the transcriptional activity of RORα/γ and RORα/γ‐mediated transactivation of BMAL1,^171^ which is a positive regulator of the clock system feedback loop. Additionally, tangeretin, a methoxylated analog of nobiletin but not a nonmethoxylated derivative such as naringin or naringenin, was also identified as a clock amplitude enhancer,^171^ which strongly supported the previously reported protective effect of tangeretin against metabolic disorders.^172^ Collectively, these natural polymethoxyflavones serve as clock amplitude enhancers that activate RORs and protect against metabolic syndrome in a clock‐dependent manner.

## 4 CLINICAL TRIALS OF NATURAL PRODUCTS FOR THE TREATMENT OF METABOLIC DISORDERS

The excellent preclinical efficacy of these natural products in treating metabolic disorders has also prompted researchers to further explore their clinical efficacy. Among the 19 natural products mentioned above, only a few of natural compounds have been tested in clinical trials for the treatment of metabolic disorders (Table 1). For example: (1) A randomized, double‐blind, and placebo‐controlled trial showed that oral supplementation of baicalin (500 mg/day for 12 weeks) obviously reduced the blood levels of triglycerides, total cholesterol, and LDL‐cholesterol in the patients with coronary artery disease.^173^ Furthermore, another randomized double‐blind trial showed oral administration of baicalin (1500 mg/day) combined with intravenous infusion administration of α‐lipoic acid (500 mg/day) for 8 weeks obviously alleviated oxidative stress and inflammatory injury in the patients with diabetic peripheral neuropathy.^174^ (2) Celastrol exhibits obvious cytotoxicity and poor pharmacokinetic properties, which restrict its further clinical application.^132^ Therefore, Ozcan's group further developed a celastrol derivative named ERX‐1000, which was identified as a first‐in‐class leptin sensitizer in preclinical obesity animal models. Preliminary clinical data obtained from a 4‐week treatment period showed that ERX‐1000 could inhibit body weight gain in obese humans (NCT04890873, https://clinicaltrials.gov/).^175^ However, the metabolic benefits of ERX‐1000 still require to be further evaluated by large‐scale randomized double‐blind controlled trials. (3) A random crossover trial showed that administration of ginsenoside Rb1 and Rg1 (41 mg/day for 2 weeks) could decrease blood levels of total cholesterol and triglyceride, and increase the mRNA expression of PPARγ in mononuclear macrophages from T2D patients.^176^ However, several clinical studies did not achieve the desired results. Chang et al.^177^ reported that oral administration of Rb1 (1 ng/kg) did not exhibit an obvious improvement on the levels of circulating glucose and insulin levels during a resistance training recovery. Thus, more clinical data are needed to address this contradiction. (4) A randomized, double‐blind, and placebo‐controlled clinical study demonstrated that oral administration of a nutraceutical product Diabetinol^®^ capsule rich in nobiletin (49%) and its analogue tangeretin (13%), at dose of 1050 mg/day for 24 weeks, could reduce the blood levels of hemoglobin A1c, LDL, and total cholesterol, as well as improve oral glucose tolerance test and 2‐h postprandial glucose profiles,^178^ which may contribute to the adjunctive therapy of T2D. However, the metabolic benefits of nobiletin still needs further clinical test.

**TABLE 1:** The clinical efficacy and safety assessment of natural products on metabolic disorders.

Classification	Compounds and administration route	Dose administered	Condition or disease	Outcome	References
Clinical efficacy	Baicalin, oral administration	500 mg/day for 12 weeks	Coronary artery disease	Decreased blood levels of triglycerides, total cholesterol, and LDL‐cholesterol	^173^
	Oral treatment of baicalin plus intravenous infusion of α‐lipoic acid	1500 mg/day and 500 mg/day for 8 weeks	Diabetic peripheral neuropathy	Decreased serum levels of SOD, malonaldehyde, TNF‐α, IL‐6, and C‐reactive protein; increased the motor conduction velocity and sensory conduction velocity, velocities of the tibial nerve and common peroneal nerve	^174^
	Celastrol analogue ERX‐1000, oral administration	4 and 8 mg/day for 4 weeks	Obesity	Reduced body weight	^175^
	Ginsenoside Rb1 and Rg1, intravenous infusion	41 mg/day for 2 weeks	T2D	Diminished total cholesterol and triglyceride levels; decreased blood glucose level, but without statistical significance	^176^
	Ginsenoside Rb1, oral administration	1 ng/kg/day for 5 days	Gymnasts were challenged by a lower‐limb resistance exercise	No significant effect on circulating glucose and insulin levels, but enhanced sympathetic nervous activity	^177^
	Diabetinol^®^ capsule containing 49% nobiletin, oral administration	1050 mg/day for 24 weeks	T2D	Reduced the blood levels of hemoglobin A1c, LDL, and total cholesterol, improved oral glucose tolerance test and 2‐h postprandial glucose profiles	^178^
Clinical safety	Baicalein, oral administration	100–2800 mg for single administration	Healthy volunteers	Safe and well tolerated by healthy subjects, and no signs of toxicity in the liver or kidney	^179^
	Red ginseng extract, oral administration	3 g/day for 15 days	Healthy volunteers	Well tolerated by healthy subjects, did not affect body temperature and blood pressure	^180^

Furthermore, among these 19 natural products, only two of them have been evaluated for their safety in human beings (Table 1). For instance, (1) single oral administration of baicalein, the aglycone of baicalin, at doses of 100−2800 mg was safe and well tolerated by healthy volunteers. Clinical laboratory assessments showed no signs of toxicity in the liver and kidney.^179^ (2) Repeated oral administration of high‐dose ginseng extract (3 g/day) for 15 days was well tolerated and did not induce significant changes in body temperature and blood pressure, and not accompanied by the accumulation of Rb1 and its metabolite, suggesting the relatively high safety of Rb1.^180^ However, the long‐term safety of these natural products still requires further evaluation.

Nonetheless, the efficacy of most other natural products has been conducted on animals rather than in clinical intervention, which thus requires further clinical validation. Moreover, adequate subject numbers and balanced sex ratio are important for the objective results of clinical trials. Finally, the existing human clinical trials of natural products are still emphasized on the detection of simple biomarkers, which lacks multi‐indicator comprehensive evaluation system associated with the functional targets of natural products.

## 5 CONCLUSION AND PROSPECTS

The fundamental pathophysiological mechanisms of metabolic disorders are impaired insulin signaling, hyperlipidemia, the inflammatory response, and oxidative stress.^14^ Recently, many natural products have been shown to alleviate metabolic disorders and complications in different preclinical models.^26^, ^27^ Some of these natural products have been modified to chemical probes to identify their functional target proteins. In this review, we summarize a total of 19 natural products (Figure 3) and their molecular modes of action against metabolic disorders (Table 2), which include (1) retaining insulin‐mediated PI3K–Akt signaling to alleviate insulin resistance by inhibiting negative regulators of the PI3K–Akt pathway (including PAQR3) or activating upstream proteins involved in insulin signaling (such as INSR and DGKQ); (2) reducing glycolipid biosynthesis to enhance insulin sensitivity by disrupting CREB–CRTC2 interaction or inhibiting SREBP‐mediated pathways in a SCAP‐ or HSP90‐dependent manner; (3) increasing lipid breakdown and energy consumption to improve metabolic disorders by activating CPT1A‐mediated FAO, activating the Dlat‐mediated AMPK–PGC1α–UPC1 thermogenesis axis, or enhancing TFEB‐mediated autophagy‐lysosomal lipid degradation; (4) blocking metabolic inflammation to attenuate metabolic disorders through the inhibition of Gal‐1‐, CAP1‐, Nur77‐, and GRP78‐mediated proinflammatory pathways; suppressing HSP90‐ or NEK7‐mediated NLRP3 inflammasome activation; or activating the Nrf2 antioxidant axis; and (5) other mechanisms, such as inhibiting PTP1B‐mediated leptin resistance and promoting RORs‐mediated activation of circadian rhythms. The identification of targets of these natural products not only reveals multifarious novel protein targets associated with the pathogenesis of metabolic disorders but also reveals structurally diverse lead compounds, thus contributing to the development of natural product‐based first‐in‐class therapeutics against metabolic disorders.

**TABLE 2:** Direct functional targets of natural products and their protective mechanisms against metabolic disorders.

Compounds	Tested models	Tested doses	Direct targets	Binding sites	Functions	References
Atractylenolide II (1)	PA‐treated HepG2 cells DIO mice and ob/ob mice	5, 10, and 20 µM 30 and 60 mg/kg	DGKQ	Ser193 Cys204 Ser241 Ala496 Phe497 His498	Allosterically activates DGKQ, reduces the hepatic sn‐1,2‐DAG levels, deactivates PKCε activity, thereby improving insulin resistance Activates DGKQ–AMPK–PGC1α–UCP‐1 signaling, thereby promoting weight loss in adipose tissue	^91^
Gentiopicroside (2)	PA‐treated HepG2 cells STZ/HFD‐induced diabetic mice	20, 40, and 80 µM 25, 50, and 100 mg/kg	PAQR3	Leu40 Asp42 Glu69 Tyr125 Ser129	Inhibits the interaction between PAQR3 and the PI3K catalytic subunit PI3K catalytic subunit p110α to restore the PI3K–Akt signaling axis and enhance insulin sensitivity	^94^
SAMC (3)	Ethanol/PA‐stimulated AML‐12 cells Ethanol‐induced liver injury mice	250 µM 300 mg/kg	INSR	Arg1164 Lys1182 Asp1183 Met1171 Met1176 Phe1186	Binds to INSR, thereby disturbing the interaction between of INSR and GRB14 to maintain insulin‐mediated Akt–GSK3β signaling	^100^
Betulin (4)	Sterol‐depletion CRL‐1601 cells DIO mice HFD‐fed LDLR^−/−^ mice	1, 3, and 6 µg/mL 30 mg/kg	SCAP	–	Physically interacts with SCAP, thereby promoting the association between SCAP and INSIG1, and inhibiting SREBP maturation and SREBP‐mediated de novo lipid synthesis	^103^
Lycorine (5)	Sterol‐depletion HL‐7702 cells DIO mice	5, 10, and 20 µM 15 and 30 mg/kg	SCAP	Tyr793 Ala1029	Directly binds to SCAP and then leads to the dissociation of SCAP‐SREBFs from INSIG1, which results in the disassociation of SCAP from the ER, thereby triggering SQSTM1‐mediated autophagy‐independent lysosomal degradation of SCAP and ubiquitylation and proteasomal degradation of SREBPs	^108^
Corylin (6)	Sterol‐depletion HL‐7702 cells DIO mice	1.5, 3, and 6 µg/mL 15 and 30 mg/kg	HSP90β	Trp312 Asn375 Asn436	Directly binds to HSP90β, thereby partially inhibiting Akt activity at Thr308 site and specifically promoted the ubiquitination and proteasomal degradation of mature form of SREBPs	^110^
Artepillin C (7)	Glucagon‐treated mouse primary hepatocytes db/db mice DIO mice	1, 5, or 50 µM 10 and 20 mg/kg	CREB	–	Directly binds to CREB and inhibits CREB–CRTC2 interaction, thereby suppressing CREB/CRTC2‐mediated gluconeogenic and SREBP transcriptions	^112^
Baicalin (8)	HFA‐treated HeLa cells DIO mice	100 µM 400 mg/kg	CPT1A	Lys286 Ile291 Glu309 His327	Directly binds to and allosterically activates hepatic CPT1A, thereby accelerating FAO	^118^
Hyperforin (9)	C3H10T1/2‐derived adipocytes ob/ob mice and DIO mice	5 µM 2.5 mg/kg	Dlat	Ser516 Arg549 Asn567	Directly binds to Dlat and upregulates its protein abundance, thereby activating Dlat–AMPK–PGC1α–UCP1 axis	^123^
Nuciferine (10)	PA‐exposed HepG2 cells HFD‐induced MAFLD mice	100 µM HFD containing 0.01 or 0.03% nuciferine	HBXIP	Thr36 Glu40 His41 Val44 Ile45 Gly72 Ile74 His87	Interacts with HBXIP and impairs the interaction of the Ragulator complex with Rag GTPases, thereby suppressing lysosomal localization and activation of mTORC1, which activates TFEB‐mediated autophagy–lysosomal pathway	^126^
Bruceine A (11)	HG‐stressed HBZY‐1 cells db/db mice	10, 20, and 50 nM 0.5 and 2 mg/kg	Gla‐1	His44 Arg48	Directly binds to Gla‐1 and disrupts the interaction between Gla‐1 and RACK1, thereby suppressing Gal‐1‐mediated activation of NF‐κB, JNK/p38 MAPK, and Akt pathways	^131^
Celastrol (12)	Resistin‐treated THP1 cells HFD‐fed mice	10, 30, and 100 nM 0.75, 1.5, and 3 mg/kg	CAP1	–	Binds to CAP1 and inhibits the interaction between CAP1 and resistin, which blocks cAMP–PKA–NF‐κB signaling	^136^
	TNF‐α‐treated HepG2 cells DIO mice	1 µM 0.1 mg/kg	Nur77	–	Directly binds to Nur77 and promotes its migration from the nucleus to mitochondria, where it is ubiquitinated by TRAF2. Ubiquitinated Nur77 then interacts with SQSTM1, resulting in mitophagy	^137^
	PA‐treated RAW264.7 cells DIO mice	1 µM 5 and 7.5 mg/kg	GRP78	Cys41	Binds to GRP78 via covalently modifying Cys41, diminishing the chaperone activity of GRP78 and ER stress	^143^
Oridonin (13)	LPS/nigericin‐treated BMDMs HFD‐fed mice	0.5, 1, and 2 µM 3 mg/kg	NLRP3	Cys279	Forms a covalent bond with Cys279 of NLRP3 to block the interaction between NLRP3 and NEK7, thereby inhibiting NLRP3 inflammasome assembly and activation	^148^
Carnosol (14)	LPS/nigericin‐treated BMDMs MCD‐induced NASH mice	5, 10, and 20 µM 40 mg/kg	HSP90	–	Binds to HSP90 and inhibits its ATPase activity, thereby disrupting the association between NLRP3 and HSP90, and inhibiting NLRP3 inflammasome assembly and activation	^150^
Echinatin (15)	LPS/nigericin‐ or LPS/ATP‐treated BMDMs MCD‐induced NASH mice	10, 20, and 40 µM 20 and 40 mg/kg	HSP90	–	Binds to HSP90 and inhibits its ATPase activity, thereby disrupting the interaction between NLRP3 and HSP90, and inhibiting NLRP3 inflammasome assembly and activation	^151^
Licochalcone B (16)	LPS/nigericin‐ or LPS/ATP‐treated BMDMs MCD‐induced NASH mice	5, 10, 20, and 40 µM 20 and 40 mg/kg	NEK7	–	Directly binds to NEK7 and inhibits the interaction between NLRP3 and NEK7, thus suppressing NLRP3 inflammasome assembly and activation	^154^
Schisandrin B (17)	HG‐exposed H9c2 cells db/db mice STZ‐induced diabetic mice	2.5, 5, and 10 µM 20 and 40 mg/kg	MyD88	Thr272 Arg288	Directly interacts with MyD88, thereby inhibiting TLR4–MyD88 interaction and MyD88‐dependent MAPK/NF‐κB proinflammatory pathway	^158^
Ginsenoside Rb1 (18)	HG/ox‐LDL‐treated EA.hy926 cells STZ/HFD‐fed ApoE^−/−^ mice	3, 10, and 30 µM 50 mg/kg	Keap1	Tyr525 Arg415 Val467	Directly binds to Keap1 and promoted its ubiquitination and proteasomal degradation by recruiting SYVN1, leading to activate Nrf2–PGC1α signaling	^167^
			p47^phox^	Thr4 Gln33	Binds to p47^phox^ and reduces its phosphorylation and membrane translocation, thereby disrupting the assembly of the NOX2 complex	^167^
Nobiletin (19)	Hepa1‐6 cells DIO mice db/db mice	1, 3.3, and 10 µM 200 mg/kg	RORα/γ	–	Bind to the ligand binding domain of RORα/γ, thereby enhancing the transcriptional activity of RORα/γ and RORα/γ‐mediated transactivation of BMAL1, ultimately enhancing circadian rhythms	^171^

Although significant advances in identifying targets of natural products have been reported, there are several limitations in these studies. (1) The binding modes between natural products and their protein targets are largely unclear. This poses a significant obstacle for further structure‐based optimization of lead compounds for better potency and safety. Direct evidence from the structures of protein–compound complexes should clarify the binding sites and binding modes and allow structural optimization and structure–activity relationship analysis of these natural products. (2) Most of these natural products exhibit poor absorption, distribution, metabolism, excretion, water solubility, or potential toxicity, which prevents clinical testing.^181^ Nanopackaging drug delivery techniques may be applied to increase solubility and bioavailability, as well as reduce usage dose and toxicity.^182^ This approach may allow natural products to undergo clinical trials without further structural optimization. (3) As metabolic disorders are defined as a class of chronic disease, they require long‐term medication. Therefore, some of the strategies used above may elicit undesirable consequences, for example, long‐term activation of insulin signaling almost always results in membrane clearance of GLUT4.^183^ The human body, on the other hand, often deactivates an overstimulated pathway after a period to avoid harm. Therefore, it is necessary to evaluate the safety and effectiveness/desensitization period of these potential natural products before they can be used in clinical applications. (4) Several of the strategies mentioned above, for example, inhibiting lipid synthesis, may require further clarification. While inhibiting lipid synthesis reduces the level of local and circulating lipids, building materials for lipids, such as glucose and glycerol, can accumulate and cause undesirable side effects. This mechanism is somewhat “out of the frying pan and into the fire” and may not fundamentally explain the actual action of the related natural products.

## AUTHOR CONTRIBUTIONS

Xiaofei Shen and Qingxiang Sun conceptualized the manuscript and drafted the initial manuscript. Xiaofei Shen and Hongling Yang prepared/improved the figures. Yang Yang, Xianjun Zhu, and Qingxiang Sun offered direction and guidance, and provided relevant resources. All authors revised the manuscript and approved the final manuscript.

## CONFLICT OF INTEREST STATEMENT

The authors declare that there are no conflict of interest.

## ETHICS STATEMENT

Not applicable.

## Data Availability

Not applicable.
